# Public attitudes to, and perceived impacts of 20mph (32 km/h) speed limits in Edinburgh: An exploratory study using the Speed Limits Perceptions Survey (SLiPS)

**DOI:** 10.1016/j.trf.2021.11.022

**Published:** 2022-01

**Authors:** Andrew James Williams, Jillian Manner, Glenna Nightingale, Kieran Turner, Paul Kelly, Graham Baker, Claire Cleland, Ruth Hunter, Ruth Jepson

**Affiliations:** a Population and Behavioural Science, School of Medicine, University of St Andrews, Room 219, Medical and Biological Sciences Building, North Haugh, St Andrews, Fife KY16 9TF, United Kingdom; b Scottish Collaboration for Public Health Research and Policy (SCPHRP), School of Health in Social Science, University of Edinburgh, 5 Forrest Hill, Edinburgh EH1 2QL, United Kingdom; c Physical Activity for Health Research Centre (PAHRC), Institute for Sport, PE and Health Sciences, University of Edinburgh, Moray House School of Education and Sport, Edinburgh EH8 8AQ, United Kingdom; d Centre for Public Health, School of Medicine, Dentistry and Biomedical Sciences, Queen’s University Belfast, Institute of Clinical Sciences, Royal Victoria Hospital, Belfast BT12 6BA, United Kingdom

**Keywords:** Policy, Transport, 20mph(32km/h), Public perceptions, Walking, Cycling

## Abstract

Between 2016 and 2018 a policy was implemented to increase the proportion of 20mph (32km/h) streets in Edinburgh, UK from approximately 50% to 80%, providing the opportunity to evaluate how behaviour and public perceptions change over time. This is important as negative public responses have been reported to limit the implementation of transport policies and may reduce the effectiveness of the policy. The Speed Limits Perception Survey (SLiPS) was developed to assess changes in public perceptions from baseline to 6 and 12 months post-implementation. We collected 3,485 individual responses to the survey, 64.6% (n = 2,253) of which included complete perceptions data. Using exploratory factor analysis, the following perception factors were identified: i) *Detraction and resistance*, ii) *Support*, iii) *Rule following*, iv) *Child safety*, and v) *Walking safety*. Following the 20mph implementation at 6–12 months: *Support* (ii) and *Rule following* (iii) had increased; *Detraction and resistance* (i) had decreased; and *Child safety* (iv) and *Walking safety* (v) had not changed significantly. These findings indicate that the public in Edinburgh became more positive towards the policy once it was implemented. However, more extensive policy or ongoing communication of the safety benefits of 20mph limits are needed to increase perceptions of safety that might lead to increased walking and cycling. Future research should aim to understand how those implementing speed limit interventions can positively influence public perceptions and how public perceptions about speed limits influence behaviour.

## Highlights

• The Speed Limits Perception Survey (SLiPS) measures perceptions of 20mph limits.
• Support for 20mph limits in Edinburgh rose after an increase in 20mph streets.
• Perceived walking or child safety did not change after 20mph limit implementation.
• Further research will examine whether these changes in perception affect behaviour.
• Studies of changes in perceptions will support transport policy implementation.

## 1 Introduction

Public responses to policy proposals can have a substantial influence on the political will to implement the policy. Negative public response has previously been reported to limit the implementation of transport policy (Aldred, 2019, Ison & Wall, 2002, Reynolds et al., 2020). Yet driving-related transport policies, such as speed limits, have been advocated as public health interventions (Sauter & Huettenmoser, 2008, British Academy, 2014). In the field of public health there is a greater burden of proof required to implement interventions that limit choice (Nuffield Council on Bioethics, 2007). However, interventions which make it easier to take the beneficial action are also recognised as delivering more population impact for lower levels of individual effort (Frieden, 2010). From the perspective that drivers have priority on the road, speed limit interventions may be viewed as limiting driver choice. Alternatively, when prioritising all road users speed limit interventions could be considered to be increasing choice for more groups within society (Sauter & Huettenmoser, 2008). Transport policies like seatbelts and cycle helmets initially faced resistance, but have become more widely adopted over time, raising questions around the balance of interests in making transport policy (Farooq, Ahmed, & Saeed, 2021). Subsequently, research into the links between perceptions of transport policy and behaviour is needed.

Beliefs or perceptions of the outcome of a behaviour have long been thought of as determining the behavioural action taken (Becker, 1974). Krabbenborg, Molin, Annema, and van Wee (2020) used Q-methodology to explore the framing of road pricing policies (e.g. congestion charges) by members of the public in the Netherlands. The four framings they identified were: ‘*the polluter should pay’*, ‘*focus on fair alternatives’*, ‘*what’s in it for me?*’, and ‘*don’t interfere’*, only the first of which supported the need for road pricing policy. Krabbenborg et al. (2020) ‘*found that factors such as equity, institutional trust, environmental beliefs, self-interest and belief in effectiveness*’ were important contributors to the framing of road pricing policy. The relationships between road users were highlighted in the analysis of Transport for London’s survey from the People and Places study related to ‘mini-Holland’ interventions promoting cycling in London conducted by Aldred (2019). They found that drivers placed the blame for issues like congestion and pollution on cyclists and vice versa, with the externalities of driving accepted but the externalities of cycling contested.

Another example relates to the implementation of reduced speed limit interventions. In Great Britain a national speed limit of 30mph (48 km/h) applies on all roads with street lighting unless there are signs to indicate another speed limit (GOV.UK, no date). Twenty miles per hour (mph, 32km/h) speed limits began to be implemented in 1990 following guidelines from the Department for Transport (ROSPA, 2020). Initially 20mph speed limits were introduced around schools, and in 1999 new policy meant that local governments could introduce 20mph speed limits without needing to seek permission from the national government. Initially these new reduced speed limits required the installation of traffic calming measures (20mph speed zones), but from 2011 they could be introduced using road signs and markings alone (20mph speed limits). Tapp, Nancarrow, and Davis (2015) surveyed the British public about their support and compliance with 20mph (32 km/h) speed limits. They identified higher levels of support for 20mph limits rather than compliance with them, noting both supporters who reported not complying and opponents who reported complying with the speed limits. Not wishing to break the law was associated with being an opponent-complier, while supporter-non-compliers tended to report themselves as trusting their own judgement and viewed the speed limits as being for other people. Together these papers demonstrate the complexity of travel behaviour and the related perceptions, each study identifying broader issues that determined attitudes to transport policies.

The growing recognition of this complexity in other arenas like health has led to calls for more practice-based evidence through applying diverse evaluation methods such as natural experiments (Reed et al., 2018, Ogilvie et al., 2020). This approach may provide insights into the mechanisms through which policy changes lead to changes in outcomes. It may also be possible to identify specific perceptions on which efforts might need to focus to foster greater public support for policy. Consequently, the city-wide implementation of a 20mph speed limit intervention in Edinburgh, United Kingdom (UK) from 2016 to 2018 provided the opportunity for the evaluation of a natural experiment and specifically, the identification of public attitudes to, and perceived impacts of a 20mph speed limit intervention.

### 1.1 Edinburgh city 2016–2018 20mph (32 km/h) speed limit intervention

Edinburgh has been the capital city of Scotland since the 15th Century. It is currently estimated to have a population around 524,930, living over an area of 263 km^2^ resulting in a population density of around 1,993 persons per km^2^ (City of Edinburgh Council, 2020, National Records of Scotland, 2019). This is around half the population density of other British cities like Bristol or Liverpool (City of Edinburgh Council, 2020). Around 74.6% of the working age population of the city are in work, with financial and insurance activities contributing the largest proportion of the city’s gross value added (City of Edinburgh Council, 2020). The city surrounds Edinburgh Castle and includes buildings from a wide range of historical periods. Subsequently, the majority of the city’s road network is single lane. Nineteen percent of the population walk to work, with a further 34% taking the bus or cycling to work (City of Edinburgh Council, 2020).

Prior to 2016 around 50% of the city’s road network had 20mph (32km/h) speed limits, most of these roads were in residential areas. A city-wide speed limit order was introduced to increase the proportion of streets with lower 20mph speed limits up to 80%, including a number of main roads (Transport and Environment Committee, 2019). The process leading up to the city-wide adoption of 20mph speed limit in Edinburgh is described by Milton et al. (2021). An arterial network of roads remained at speed limits higher than 20mph to support efficient travel where necessary (Transport and Environment Committee, 2019). The logistical difficulties of implementing 20mph (32km/h) speed limits across a whole city simultaneously meant that a phased approach was taken, converting different regions of the city at different times between 2016 and 2018. The seven implementation regions were defined specifically for this policy change and named after their compass position around the city centre.

Alongside the city-wide speed limit order legislation and the road signs and markings, there were awareness raising, educational and enforcement activities. Adverts were put up in public spaces, including on the outside of buses. Leaflets, a website (https://www.edinburgh.gov.uk/20mph-edinburgh) and media coverage promoted and explained the new policy. Advert and leaflet campaigns were undertaken in each implementation region in the weeks leading up to the 20mph speed limit intervention being implemented. It was initially agreed with the police not to issue fines for exceeding the new 20mph speed limits, but warning letters. Targeted speed camera campaigns were later introduced on roads where many warnings were being issued. Specific data on the number of enforcement activities has not been available to the research team. However, Nightingale, et al. (under review) have documented the decreases in vehicle speeds in the city, and Popov et al. (2021) have documented a subsequent reduction in collisions and casualties.

The aim of the present study was to examine how public perceptions of a transport policy (20mph (32km/h) speed limits) change, following implementation of the policy. The objectives of the current study were to:

(1) Describe the public attitudes to, and perceived impacts of 20mph (32km/h) speed limit interventions prior to city-wide implementation of 20mph (32km/h) speed limits.
(2) Examine whether and how these public attitudes and perceived impacts change at 6 months and 12 months following city-wide implementation of 20mph (32km/h) speed limits.
(3) Identify 20mph (32km/h) speed limit perception factors using exploratory factor analysis to account for correlation between attitudes and perceived impacts.
(4) Examine how these perception factors are associated with participant socio-demographics and travel behaviours.
(5) Analyse whether these perception factors change following the city-wide implementation of 20mph (32km/h) speed limits.

## 2 Material and methods

This study was part of the National Institute for Health Research funded ‘Is 20 plenty for health?’ study (Jepson et al., 2017). The aim of the ‘Is 20 plenty for health?’ study was to evaluate the processes up to and following the policy change and the broad public health outcomes of 20mph speed limits (commonly equated to 30km/h speed limit policies in other countries) in Edinburgh and Belfast, United Kingdom (UK). This was a mixed methods study, using existing data resources within a natural experimental framework where possible. Existing evidence and early qualitative work (Turner et al., 2018) suggested that public perceptions about 20mph speed limits were key antecedents of behaviour change and policy impact. Consequently, to understand and explain the impact (or lack of impact) of the policy, it was valuable to assess perceptions of the intervention and evaluate how they may alter. The current study only relates to the policy and evaluation in Edinburgh, UK.

**Fig. 1:**
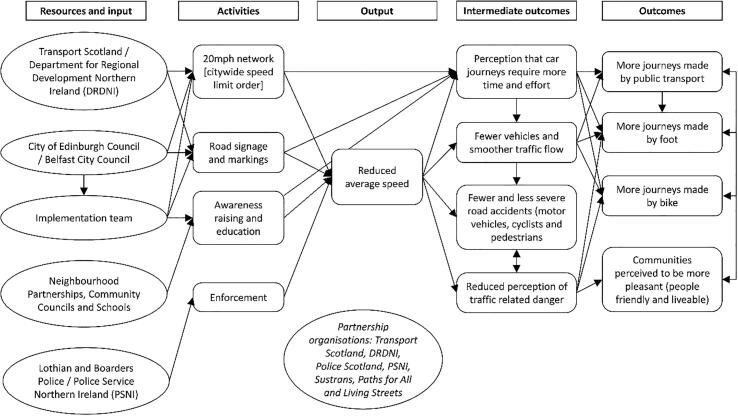
Original ‘Is 20 plenty for health?’ study logic model of the hypothesised processes and outcomes of 20mph (32 km/h) speed limits (Jepson et al., 2017).

The ‘Is 20 plenty for health?’ study was a theory driven evaluation (Milton et al., 2021). There are multiple and complex pathways through which the policy could lead to a range of outcomes. While developing the study proposal a logic model was developed to capture the expected mechanisms and outcomes. The original logic model is shown in Fig. 1, and this has been revised as further data and insights have been gathered (e.g. Turner et al., 2018). It is clear from the logic model that public perceptions were thought to be important to achieving the desired results of the intervention. The educational and awareness raising activities implemented by the City of Edinburgh Council sought to influence public perceptions. The council undertook a survey (Progressive, 2019) alongside the intervention, however this focused on safety and mode of travel and therefore it was necessary to conduct an additional survey to capture data on the breadth of perceptions related to 20mph speed limits.

A previous nationwide study of 20mph (32 km/h) speed limits by Tapp et al. (2015) also used a survey to assess perceptions, but this was intended to only be completed by drivers and did not ask questions related to all the pathways and outcomes in the ‘Is 20 plenty for health?’ logic model (Fig. 1). Additionally, the survey used by Tapp et al. (2015) was not designed to detect change, which was a principal feature of the current study. Therefore, the Tapp et al. (2015) survey was adapted for the present study into the Speed Limits Perceptions Survey (SLiPS), with the final survey given in Appendix 1. The adaptations were minor with statements being altered to be relevant to all road users, wording adjusted to account for whether the survey was taking place before or after the policy change, and statements related to safety and liveability being added. SLiPS comprises two major sections, five subsections and 53 questions:

• Participant characteristics
  o Demographics (6 questions)
  o Travel habits (8 questions)
• Perceptions
  o General attitudes to the road (8 questions)
  o Attitudes to 20mph speed limits (16 questions)
  o Perceived impacts of 20mph speed limits (15 questions)

### 2.1 Data collection

Twenty miles per hour speed limits were introduced in the first implementation region in Edinburgh on 31st July 2016, but the ‘Is 20 plenty for health?’ study was not funded until early 2017, when half of the implementation regions in the city had already been converted to 20mph speed limits. Subsequently, pre- and post-intervention perception survey data could only be collected in the remaining three implementation regions.

The first two implementation regions to be converted to 20mph after this study received funding were implemented on the same day (16th August 2017), and were adjacent to each other. The final implementation region adopted 20mph on 5th March 2018. Subsequently, there were two foci (named hereafter as Implementation regions 4 and 5, and Implementation Region 6) for data collection (the two implementation regions becoming 20mph in August 2017, and the final implementation region in March 2018), with data collected at three time points (baseline, 6 months and 12 months). We aimed to recruit 500 participants per time point, with the hope that there would be at least 300 (60%) complete responses, providing a total sample size of approximately 1,800 responses. Alongside some members of the study team, the data were collected by Undergraduate and Postgraduate students (listed in Appendix 2) from the University of Edinburgh who received training in completing the survey with participants. Three large shopping centres, a sports/leisure centre and a hospital were selected as sites with enough local footfall to support the recruitment of sufficient numbers of people from Implementation regions 4 and 5, and Implementation Region 6. Having gained permission from the relevant gatekeepers at each site, data collection at each time point took place over 2–5 days (Appendix 3) at various times of day. Data collection dates were scheduled as close as possible to the appropriate time points, subject to site availability. Baseline data collection was taken no more than 4 weeks prior to 20mph implementation in each implementation region. Follow-up data collection was conducted within 4 weeks of each implementation region’s 6 and 12 month post-implementation anniversaries.

At each site, potential participants were approached about the survey, and the study was explained to those who were interested. Participants had to be adults over 16 years of age who could provide consent and complete the survey in English. Participants who understood the purpose of the study and could provide consent, but were unable to fill out the survey on their own (e.g. if they had difficulties reading or writing) were aided in completing it. Written informed consent was collected prior to completion of the survey on paper. The participants were offered the opportunity to enter a competition to win a £100 shopping voucher. This study was reviewed and approved by the University of Edinburgh, Moray House School of Education Ethics Sub-committee (ref: 1114).

### 2.2 Data analysis

The postcodes of participants’ homes were linked to the Scottish Index of Multiple Deprivation 2016 (SIMD16) quintile and Scottish Government Urban Rural Classification 2016 of their local area (Geographic Information Science, 2016, Scottish Government, 2017). SIMD16 is a relative measure of area deprivation derived from a large number of administrative data sources across seven domains: income, employment, education, health, access to services, crime and housing (Scottish Government, 2017). While the Scottish Government classification categorises areas with populations of 125,000 or more as Large urban areas, those with populations of 10,000 to 124,999 as Other urban areas, those with populations of 3,000 to 9,999 as Small towns and those with populations below 2,999 as Rural areas (Geographic Information Science & Analysis Team, 2018). SIMD16 and the Urban Rural classification provide context to the participant responses and permit the examination of geographic and socioeconomic inequalities.

As with most questionnaires, within the SLiPS multiple questions were asked related to single underlying constructs such as safety or self-enforcement. Confirmatory factor analysis had been used during the development of the survey to test whether the survey assessed support for 20mph. In order to explore this further and reduce the number of variables, exploratory factor analysis of the complete dataset was undertaken. Having used the logic model (Fig. 1) to inform the questions included in the survey, taking an exploratory approach to the factor analysis allowed latent (potentially missed) perceptions to be revealed. As the responses from the survey were ordinal or binary, the factor analysis used a polychoric correlation matrix and promax rotation (Institute for Digital Research & Education Statistical Consulting, 2020). With the aim to explore how perceptions altered following implementation of the 20mph speed limits, the factor analysis was conducted on only baseline survey responses, and subsequently those variable loadings were used to calculate the perception factors at all three time points. Due to the focus on the baseline data, six questions that were not introduced until after the first round of baseline data had been collected, were not included in the factor analysis:

• We only need 20mph limits where safety is an issue (e.g. schools, dangerous junctions)
• We do not need 20mph limits at night when roads are quieter
• 20mph speed limits will/have led to less noise from vehicles
• 20mph speed limits will/have led to an increase in how pleasant the area is to live or work in
• 20mph speed limits will/have led to safer streets
• 20mph speed limits will/have led to more opportunities to socialise

To explore whether these additional questions meaningfully altered the perception factors identified, the follow-up data, and separately the baseline data from the final implementation region, were also analysed for perception factors. The analyses consistently identified 4 or 5 perception factors (with Eigen values great than 1 (before the elbow)). The constructs behind the perception factors did not alter markedly with the last two of the perception factors combining when only 4 rather than 5 perception factors were identified. The variables loading into each perception factor remained consistent with the loadings altering by at most ± 0.15.

The repeat cross-sectional design meant that different participants were recruited at each time point and location. A complete case approach was taken to the analysis as factor analysis requires that there are no missing data. The data from all locations were combined for the analysis, which was conducted in Stata (StataCorp, 2015) using two-tailed tests and α = 0.05. Apart from one question, all the responses were categorical, and therefore percentages were used to descriptively examine both the perceptions and the participant characteristics. Standard statistical tests (e.g. chi-squared) were used to test for significant differences between time points. To explore the impact of including different people in each survey data collection, the associations between each factor score and participant demographics and travel behaviours were tested using standard statistical tests. Subsequently, the plan was to calculate sampling weights to account for the differences in participation. These weightings would then be used within regression models to explore whether perception factors had altered irrespective of the participation. The single open-ended question in the survey asked participants to list what ‘other road safety policies would make you cycle more’. The responses were extracted and enumerated to identify frequently named policies.

## 3 Results

### 3.1 Recruitment

Details of the number of participants in the survey at each time point are provided in Table 1, with the proportion providing complete perceptions data also reported. Only in one data collection point (6 months Implementation Region 6) were complete perceptions data available for less than 60% of participants. Complete perceptions data were available for 1,018 participants at baseline, 599 participants at 6 months and 636 participants at 12 months, providing a total sample of 2,253 (64.6%). Summaries of the demographics and travel behaviours of the participants with complete perceptions data are listed in Table 2. The amount of missing data for each of the demographic and travel behaviour variables was relatively low (<10%), but higher for some of the travel behaviour questions, whether the participant worked (which might reflect social desirability bias) and SIMD16 quintile and Scottish Urban Rural classification (where some postcodes could not be matched).

**Table 1:** Survey responses and complete perceptions data rates by time point and implementation region (number and (percentage)).

	Implementation Regions 4 and 5 (16th August 2017)		Implementation Region 6 (5th March 2018)		Total	
	Total response	Complete responses (%)	Total response	Complete responses (%)	Total response	Complete responses (%)
Baseline	969	700 (72.2)	497	318 (64.0)	1,466	1,018 (69.4)
6 months	507	313 (61.7)	501	286 (57.1)	1,008	599 (59.4)
12 months	509	327 (64.2)	502	309 (61.6)	1,011	636 (62.9)
Total	1,985	1,340 (67.5)	1,500	913 (60.9)	3,485	2,253 (64.6)

**Table 2:** Participant demographics and travel behaviours.

Timepoint		Baseline	6 months	12 months
Complete sample size		1,018	599	636
**Decade of birth**	Pre-1950	15.3% (151)	13.6% (79)	9.44% (58)
	1950s	18.8% (186)	19.4% (113)	15.6% (96)
	1960s	23.5% (233)	26.8% (156)	21.6% (133)
	1970s	16.1% (159)	19.0% (111)	20.6% (127)
	1980s	12.4% (123)	11.2% (65)	13.8% (85)
	Post-1989	13.9% (138)	10.1% (59)	19.0% (117)
	Missing	28	16	20
Gender	Male	47.1% (470)	48.7% (289)	47.3% (292)
	Female	52.9% (528)	51.4% (305)	52.7% (325)
	Missing	20	5	19
Disability		6.0% (60)	5.5% (32)	4.6% (28)
	Missing	23	18	26
**Ethnic minority**	Non-White	5.0% (49)	6.3% (37)	10.0% (62)
	Missing	31	11	17
SIMD16 Quintile	(most deprived) quintile 1	9.4% (69)	8.2% (33)	5.5% (26)
	quintile 2	12.0% (88)	11.9% (48)	12.5% (59)
	quintile 3	13.0% (95)	9.6% (39)	13.1% (62)
	quintile 4	16.1% (118)	19.8% (80)	20.6% (97)
	(least deprived) quintile 5	49.5% (362)	50.6% (205)	48.3% (228)
	Missing	286	194	164
Scottish Government Urban Rural Classification 2016	Large urban areas	86.1% (630)	86.9% (352)	83.7% (395)
	Other urban areas	5.3% (39)	5.7% (23)	6.8% (32)
	Small towns	4.4% (32)	2.5% (10)	5.9% (28)
	Rural areas	4.2% (31)	4.9% (20)	3.6% (17)
	Missing	286	194	164
**Not working**		48.9% (392)	64.9% (362)	72.3% (405)
	Missing	217	41	76
**Full UK driving licence**		94.3% (957)	94.8% (565)	90.4% (571)
	Missing	3	3	4
**Driving experience**	<5 years	10.2% (95)	8.0% (43)	12.6% (68)
	5–10 years	10.1% (94)	6.7% (36)	9.4% (51)
	10–20 years	17.8% (165)	14.2% (76)	16.1% (87)
	20–40 years	44.8% (416)	50.5% (270)	46.2% (250)
	>40 years	17.1% (159)	20.6% (110)	15.7% (85)
	Missing	89	64	95
Motorcycle user		5.3% (49)	5.9% (32)	4.8% (27)
	Missing	99	59	70
**Are the roads near your home or work 20mph**	No	19.9% (201)	12.2% (72)	12.6% (79)
	Yes	75.2% (760)	84.7% (499)	84.0% (525)
	Don't know	5.0% (50)	3.1% (18)	3.4% (21)
	Missing	7	10	11
**Are you aware of any (more) plans for 20mph limits in the area where you live?**	No	45.6% (460)	53.1% (317)	54.2% (341)
	Yes	37.6% (379)	28.1% (168)	18.9% (119)
	Don't know	16.9% (170)	18.8% (112)	26.9% (169)
	Missing	9	2	7
Frequency of use of bus, train or tram	Every day	11.9% (118)	11.9% (69)	13.5% (84)
	Several times a week	21.6% (214)	22.0% (128)	23.8% (148)
	About once a week	16.8% (167)	19.1% (111)	17.5% (109)
	About once a fortnight	12.0% (119)	12.9% (75)	12.2% (76)
	About once a month	13.4% (133)	11.9% (69)	13.2% (82)
	Less than once a month	15.9% (158)	13.9% (81)	14.1% (88)
	Never	8.5% (84)	8.3% (48)	5.8% (36)
	Missing	25	18	13
**Frequency of use of a car or van**	Every day	46.4% (462)	44.5% (259)	42.0% (261)
	Several times a week	33.7% (335)	34.0% (198)	30.0% (186)
	About once a week	10.0% (99)	9.5% (55)	11.9% (74)
	Less than once a week	4.8% (48)	8.3% (48)	9.5% (59)
	Never	5.1% (51)	3.8% (22)	6.6% (41)
	Missing	23	17	15
Frequency of use of taxi or Uber	Less than once a fortnight	8.7% (81)	7.2% (39)	11.4% (66)
	About once a fortnight	9.8% (91)	9.7% (53)	9.0% (52)
	About once a month	14.3% (133)	15.8% (86)	17.8% (103)
	Less than once a month	32.3% (301)	34.5% (188)	33.2% (192)
	Never	35.1% (327)	32.8% (179)	28.7% (166)
	Missing	85	54	57
Cycling frequency	Several times a week	9.4% (89)	11.5% (64)	12.5% (74)
	Several times a month	10.6% (100)	11.7% (65)	9.8% (58)
	Less than once a month	9.1% (86)	9.9% (55)	11.8% (70)
	Never	71.0% (672)	67.0% (374)	65.9% (391)
	Missing	71	41	43
**Walking or running frequency**	Every day	34.5% (338)	42.3% (242)	41.7% (254)
	Several times a week	31.1% (304)	29.9% (171)	29.2% (178)
	About once a week	11.1% (109)	9.4% (54)	10.8% (66)
	Less than once a week	10.0% (98)	8.9% (51)	9.9% (60)
	Never	13.3% (130)	9.4% (54)	8.4% (51)
	Missing	39	27	27

Variables in **bold** vary statistically significantly across time points.

### 3.2 Participant characteristics and travel habits

Participants were mostly born in the 1950s–70s, although the proportion born from 1990 onwards was higher in the 12 months post-intervention survey. When compared to the mid-2018 population estimates for the City of Edinburgh, the survey respondents were older with more people born prior to the 1980 s in the survey sample (73.2%) compared to 54.2% of the eligible population (16 or more years of age) of the City of Edinburgh (National Records of Scotland, 2019). Across time points the gender mix and proportion of people who declared a disability did not vary significantly, with around 5–6% declaring a disability and slightly more females than males participating at each time point. The gender mix was consistent with the mid-2018 population estimates where 51.2% of the population of the City of Edinburgh council area were female, but 16.1% of the city’s population reported that their day-to-day activities were limited a little or a lot by a long-term health problem or disability (National Records of Scotland, 2019, National Records of Scotland, 2020). The proportion of people from an ethnic minority significantly increased across the survey sweeps, as did the number of people not in work. The SIMD quintile of participants home address remained consistent across time points with fewest people from quintile 1 (most deprived) and the numbers gradually increasing up to quintile 5 (least deprived). Around 85% of respondents across the sweeps lived in a Large urban area. The proportion of the participants at 12 months who held a full UK driving licence was significantly lower than at the earlier time points and the number of years of driving experience varied. The number of people reporting that the roads near their work and/or home were 20mph significantly increased from baseline to 6 months but did not change much from 6 to 12 months, which is consistent with the intervention. The frequency of car or van use was reported to go down and the frequency of walking increased. However, use of public transport, taxis or cycling did not change significantly.

### 3.3 Participant perceptions

**Fig. 2:**
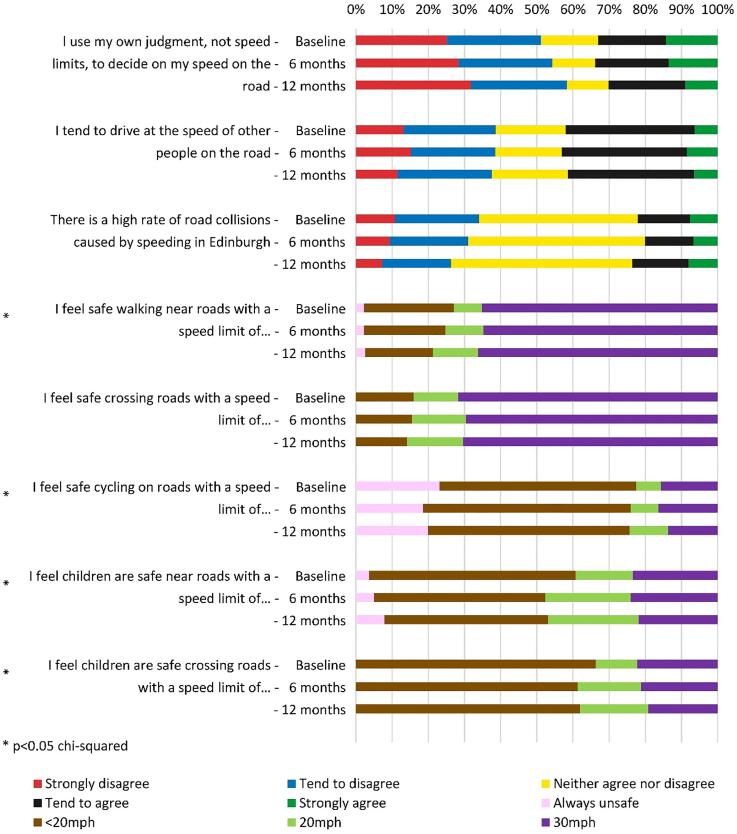
Stacked bar charts of participant general attitudes to the road at baseline, 6 months and 12 months post 20mph speed limit implementation in Edinburgh.

**Fig. 3:**
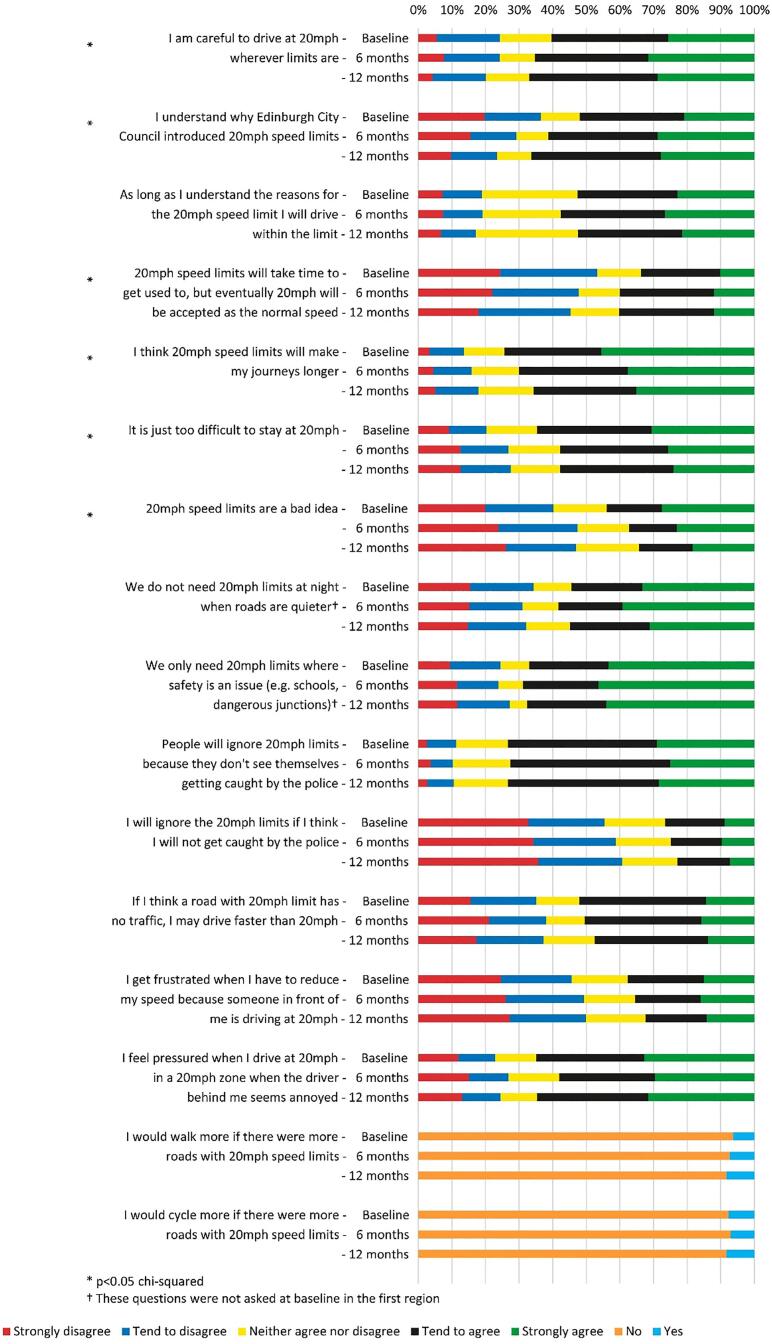
Stacked bar charts of participant attitudes to 20mph speed limits at baseline, 6 months and 12 months post 20mph speed limit implementation in Edinburgh.

**Fig. 4:**
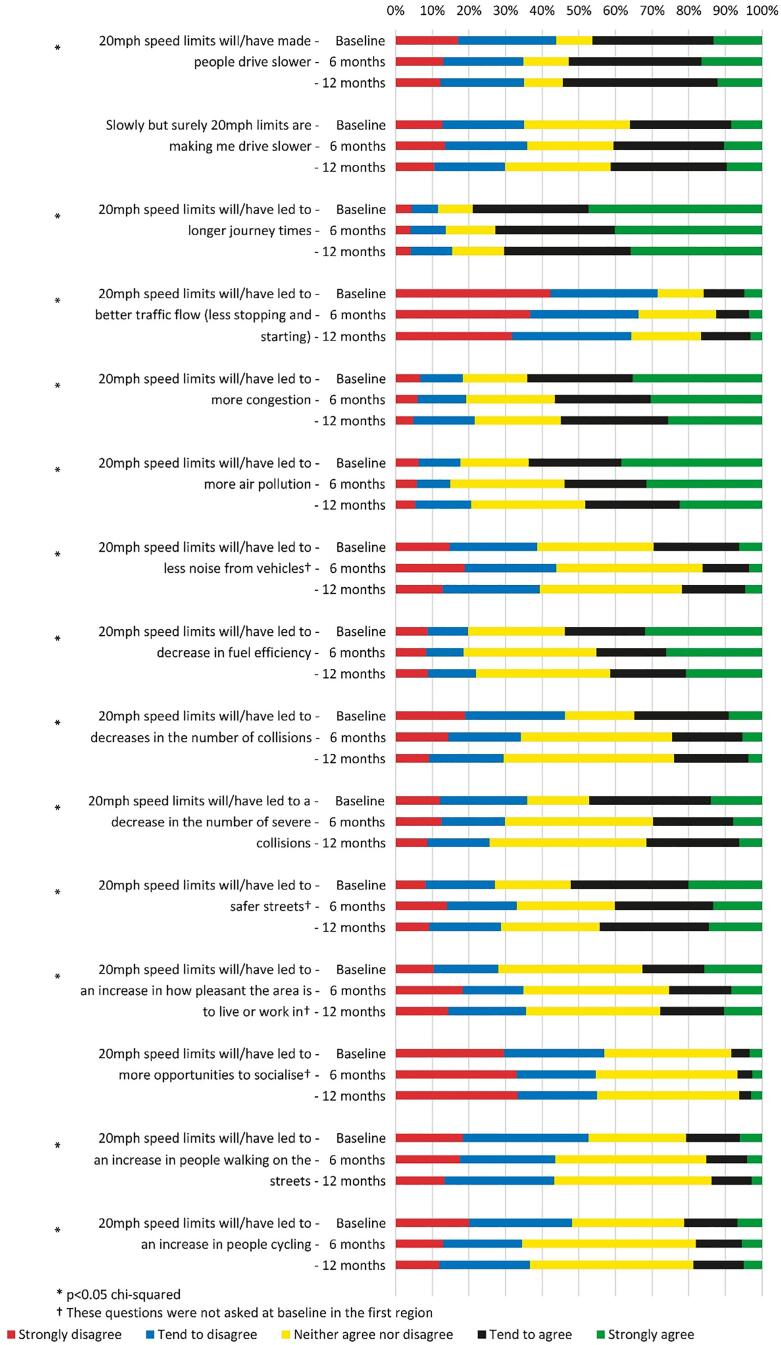
Stacked bar charts of perceived impacts of 20mph speed limits at baseline, 6 months and 12 months post 20mph speed limit implementation in Edinburgh.

Presented in Fig. 2, Fig. 3, Fig. 4 are the responses at each time point to each of the perceptions questions; general attitudes to the road in Fig. 2, attitudes to 20mph speed limits in Fig. 3, and perceived impacts in Fig. 4 (numeric responses are presented in Appendix 4). In each figure the questions have been ordered by the logic model (Fig. 1), starting from questions related to travel behaviours, then collisions and finally liveability and active travel. Responses that were found to differ significantly (chi-squared test) between time points have been marked with an asterisk (*). The greatest number of significant changes between time points were reported in relation to perceived impacts (Fig. 4), only two of which did not vary statistically significantly. However, fewest statistically significant changes were found in terms of attitudes to 20mph speed limits, which included how the individual might behave in response to the new 20mph speed limits. Attitudes towards 20mph speed limits seem to have been more polarised, while the statements about perceived impacts attracted more ambivalent responses. In general, the responses tend to indicate a shift in favour of 20mph, with reports of negative attitudes reducing and positive ones increasing. However, there was less certainty regarding the most distal impacts such as improvements in the environment and suitability for active travel (Fig. 2, Fig. 3, Fig. 4). Less than 10% of people reported that 20mph would encourage them to walk or cycle more at any time point (Fig. 3). This may reflect a distinction between behavioural intentions and the adoption of new behaviours, and challenges the idea that lower traffic speeds might encourage more walking and cycling.

### 3.4 Factor analysis

The five perception factors identified from the exploratory factor analysis of all the complete baseline responses are detailed in Appendix 5, with variable loadings less than ± 0.3 left blank (the polychoric correlation matrix is provided in Appendix 6). The possible responses for each question are listed in Fig. 2, Fig. 3, Fig. 4; ‘Strongly agree’ was coded 1 through to 5 for ‘Strongly disagree’. Therefore, positive factor loadings reflect disagreement with the statement, while negative loadings reflect agreement. Higher scores on the five statements beginning with ‘I feel’ reflect a perception of less precaution, and that the respondent felt the activity in question was safe near higher speed roads. The remaining two questions could either be answered ‘yes’ or ‘no’, with ‘no’ coded as 0 and ‘yes’ coded as 1. Based on the questions loading most markedly into each factor and how they loaded, a descriptive name has been created for each variable.

The descriptive names selected for each of the five perception factors in order are: i) *Detraction and resistance*, ii) *Support*, iii) *Rule following*, iv) *Child safety*, and v) *Walking safety*. *Support* (ii) and *Rule following* (iii) seemed to include more passive statements, while those relating to *Detraction and resistance* (i) were more active, including behaviours intended to defy the new speed limits. The *Child safety* (iv) and *Walking safety* (v) factors seemed to be independent of the 20mph speed limit policy. There were only a few instances when the same question loaded with a score greater than ± 0.3 into more than one factor. This could suggest that it would be possible to develop information or messages specifically to reduce resistance or detraction without adversely affecting support or rule following. The variable loadings were used to calculate the factors at all-time points (Appendix 5). As the factors were derived from categorical data, the calculated factors were not normally distributed and consequently all subsequent analysis uses non-parametric methods, rather than the survey weighted regression we planned.

**Fig. 5:**
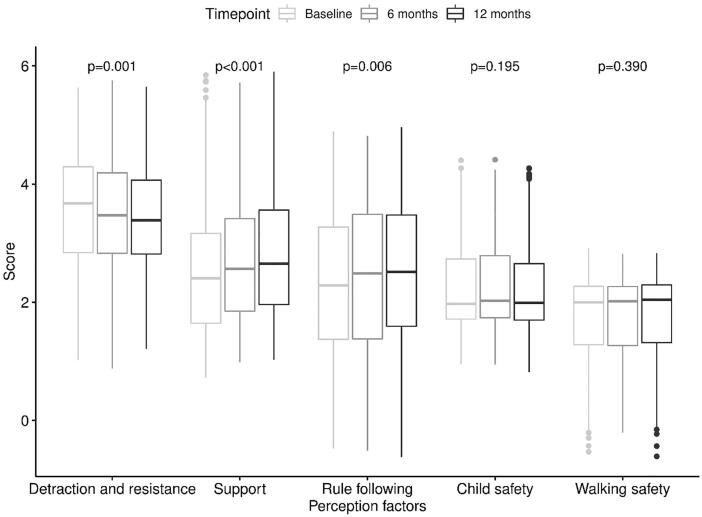
Box plots of each perception factor at baseline, 6 and 12 months p-values based on Kruskal-Wallis tests.

In Fig. 5, box plots are presented for each of the five perception factors. Reported on the figure are Kruskal-Wallis tests undertaken to compare each of the perception factors from baseline to 6 and 12 months. There were statistically significant increases in *Support* (ii) and *Rule following* (iii), a significant decrease in *Detraction and resistance* (i), and no significant change in *Child safety* (iv) or *Walking safety* (v). Given the differences in participant demographics and travel habits previously identified (Table 1), Kruskal-Wallis (categorical variables) and Mann-Whitney U (binary variables) were used to explore whether the factor scores varied significant by participant characteristic. The results of these tests have been summarised in Appendix 7. Only whether the participant was not in work was found to have no statistically significant associations with any of the factors. The identified associations are consistent with the names of the factors, with those who drove more reporting higher scores for *Detraction and resistance* (i), and those using other forms of transport more frequently reporting higher *Support* (ii) scores. However, these findings mean that any differences in factor scores identified through a direct comparison between time points (Fig. 2, Fig. 3, Fig. 4, Fig. 5) might reflect differences in survey participation.

Additional sensitivity analyses were undertaken to examine whether the changes in factor scores from baseline to 6 and 12 months differed by participant demographics and travel behaviours. The statistically significant differences identified are reported in Appendix 7 and illustrated using box plots in Appendix 8. Changes in *Detraction and resistance* (i) differed by SIMD16 quintile and awareness of plans for further 20mph limits. Changes in *Support* (ii) differed by gender, ethnic minority and being out of work. Changes in *Rule following* (iii) were found to differ by four variables: disability, motorcycle user, frequency of car or van use and walking or running frequency. Changes in *Child safety* (iv) differed by gender and being out of work, and for *Walking safety* (v) changes were different for those with different frequencies of walking or running. The size of some of the groups being compared in these sensitivity analyses were quite small (e.g. ethnic minority, motorcycle users) meaning that these findings are unreliable. *Detraction and resistance* (i) does not appear to have reduced among those living in more deprived areas compared to those from less deprived areas. *Support* (ii) increased for women after 6 months, but not until 12 months for men. However, men reported increases in perceived *Child safety* (iv) that women did not. Finally, in response to the open-ended question, participants most frequently listed cycle lanes as the road safety policy that would make them cycle more. Safety concerns in terms of driver behaviours (parking and overtaking) and road conditions (potholes) were also commonly mentioned.

## 4 Discussion

Surveying the public in Edinburgh revealed a variety of perceptions of 20mph (32km/h) speed limits, including beliefs about the impact of these speed limits. By repeating the survey 6 and 12 months after the implementation of 20mph (32km/h) speed limits it has to some extent been possible to examine whether and how these perceptions changed. While some perceptions did not change markedly, many of the assessed perceptions, both positive and negative, altered once 20mph (32km/h) speed limits were implemented. Exploratory factor analysis of the survey responses identified the following five perception factors: i) *Detraction and resistance*, ii) *Support*, iii) *Rule following*, iv) *Child safety*, and iv) *Walking safety*. Basic statistical tests indicated a statistically significant reduction in *Detraction and resistance* (i) and increases in *Support* (ii) and *Rule following* (iii), following the 20mph (32km/h) speed limit intervention in Edinburgh. Based on the statements contributing to the *Support* (ii) and *Rule following* (iii) factors, these appeared more passive perceptions, while *Detraction and resistance* (i) was more active and therefore may need to be address directly in educational and awareness raising campaigns. The increase in reports of *Rule following* (iii) align with the findings of Tapp et al. (2015), who in their UK wide survey found that opponents to 20mph (32km/h) speed limits who reported complying with the speed limits made reference to wanting to follow the rules.

People’s perceptions of child and walking safety seemed to be independent of the 20mph (32 km/h) speed limit policy and did not change significantly over time. Previous research has found that although 20mph (32km/h) speed limits might reduce the number of deaths on the road (severity of collisions), the risk of injury (number of collisions) might not change, and therefore perceptions of safety might not alter significantly (Mohit, Rosen, & Muennig, 2018). We have found reductions in both casualties and collisions following the Edinburgh City 2016–2018 20mph (32km/h) speed limit intervention (Milton et al., 2021, Popov et al., 2021). However, Elvik (2015) has identified that people tend to overestimate the risk of rare events and underestimate the risk of common events in relation to their travel behaviours. Subsequently, people’s perceptions of the risks of collisions, and consequently road safety may not change as you would hope as collisions become rarer.

This study found that the experience of a policy change alters some public perceptions of the policy. Previous research has found that information about policy effectiveness increases support for the policy, while information on policy ineffectiveness reduced support (Reynolds et al., 2018, Reynolds et al., 2020). The experience of wearing a seatbelt and not finding it uncomfortable or restrictive contributed to the wider compliance with this initially controversial policy. The discovery that your journey time did not increase, or congestion did not worsen as you feared may be an important mechanism for wider acceptance of 20mph (32km/h) speed limit interventions and improved road safety.

## 5 Strengths and limitations

While this study has brought new insights through collecting repeat surveys, there are several limitations that future studies should consider. The participants, and in some cases the locations themselves, in each sweep of data collection were different, which means that some or all of the changes in perceptions observed may be due to differences in participation. Surveying the same cohort of people repeatedly would have addressed this issue. However, repeated assessment of the same cohort increases the risk of selection and social desirability bias, anchoring and unobserved heterogeneity that would need to be accounted for in any future studies (Saeed et al., 2020). Social desirability bias may also be an issue in the present study as we only asked people to report their perceptions and behaviour rather than observing their behaviour. While a repeated cross-sectional survey can provide useful information on population-level public perceptions, the heterogeneity between the samples at each time point mean that our findings are not conclusive and further research is needed.

The attitudes evaluated in this study are likely to have been fairly ingrained as 50% of the streets in Edinburgh had 20mph speed limits prior to the recent policy change. Furthermore, by the time the ‘Is 20 plenty for health?’ study started, the 20mph intervention had been implemented in four of the seven implementation regions across the city of Edinburgh. The lack of routine data on these outcomes meant that baseline data collection for this study took place when approximately half the city had received the intervention. It is likely that our respondents had been exposed to the awareness campaigns and any effects of 20mph (public perceptions, media reports, changes in speed, etc.) at a city level, even before the intervention arrived in the implementation region in which they lived or worked. As such, larger (and perhaps more significant) changes in resistance, for example, may be seen in locations when “true” or “uncontaminated” baseline data can be collected, though the effects of national and international discourse on 20mph would still need to be considered.

Reported in this paper are a large number of statistical hypothesis tests for the sample size (n = 2,253), increasing the likelihood of finding false positive associations, further emphasising that these are exploratory findings that need to be tested further. In addition, the climate emergency is increasing the public dialogue around polluting activities including transport, which may be producing a shift towards more favourable attitudes to speed limit reducing interventions. It is unlikely that the perceptions assessed change seasonally or within any smaller time frame in the absence of an intervention, lending some validity to the changes observed. The findings of the sensitivity analyses that changes in perception may have differed among marginalised groups indicate that future research focusing on these populations would be valuable.

The development of a logic model, providing a theory on which to base the SLiPS, is a strength of the present study and other studies into transport policies (Elvik, 2015). However, previous studies into perceptions related to transport policy tended to have relied upon a single sweep of data collection, either before or after the implementation of a policy (Aldred, 2019, Ison & Wall, 2002, Krabbenborg et al., 2020, Tapp et al., 2015). Including pre and post implementation surveys has allowed us to examine which and how perceptions change, which is an important contribution to both transport and public health policy research (Aldred, 2019, Ison & Wall, 2002, Reynolds et al., 2018, Reynolds et al., 2020).

### 5.1 Implications

Negative public perceptions have been reported to lead to transport interventions being curtailed (Aldred, 2019, Ison & Wall, 2002, Tapp et al., 2015). Using the SLiPS it has been possible to assess changes in public perceptions which may be due to a speed limit intervention. Consequently, researchers need to work with policy makers to build the evidence base around changes in perceptions alongside the implementation of transport policies. Further validation of the SLiPS is necessary, but it may be useful for those seeking to learn about the perceptions of their population to current or future plans for speed limit interventions. Perceptions of speed limit interventions are likely to vary within (e.g. urban compared to rural areas, different cities) and between countries, and the SLiPS could be used to help document these differences by providing information on readiness for speed limit interventions, supporting policy decisions.

We need to understand more about public attitudes to interventions, how they determine behaviours, if at all, and whether they change in response to experiencing the intervention. Policy makers and politicians may be overly sensitive to public opinions, and therefore limiting the implementation of interventions that could improve public safety and health. Subsequently, there is a need for more studies like the ‘Is 20 plenty for health?’ study that explore both behaviours and perceptions. This will provide insights into which perceptions should be influencing policy decisions, and which perceptions would be the most effective focus for educational campaigns. Studies that observe driving behaviour alongside collecting perceptions data might be quite intrusive. However, mobile phones and devices like those used in telematic car insurance policies may permit future studies to collect these data (Guillen, Nielsen, Ayuso, & Pérez-Marín, 2019). Studies exploring perceptions among minority groups including users of the road who use motorcycles or less common means of transport are needed.

Reduced speed limit interventions are often promoted as increasing road safety (Sauter & Huettenmoser, 2008, British Academy, 2014), however, we did not detect significant changes in perceptions of walking or child safety following the Edinburgh 20mph (32km/h) speed limit intervention. Popov et al. (2021) have identified reductions in collisions and casualties in Edinburgh following the 20mph (32km/h) speed limit intervention. Future studies with longer follow-up periods may detect perceptions of safety changing beyond 12 months. However, the distinction between the objective level of risk and how people perceive and act on risks described by Elvik (2015) means that those implementing speed limit interventions may need to run campaigns following the policy change to communicate how the risk of collisions and casualties has changed. This is likely to be particularly important for achieving increases in active travel following speed limit interventions where people often cite concerns about safety as a deterrent from walking or cycling.

## 6 Conclusions

Levels of public support and resistance to 20mph (32km/h) speed limits in Edinburgh appear to have changed in favour of the policy in the three implementation regions receiving the intervention that were investigated in this study, while perceptions of safety did not change. In most societies driving is seen as a necessity, with the built environment currently designed around car use, while cycling is often seen as a luxury for the wealthy or a cheaper transport option for those experiencing poverty. Therefore, policies that affect people’s ‘freedom’ to drive attract a lot of resistance. Although, this resistance might influence a politician’s approval rating, should that be a sufficient reason to limit policy that impacts on public safety and health? Future research needs to validate the SLiPS and test how perceptions change in different contexts, especially among minority groups. However, evidence of the benefits from environments designed for all road users is growing. It has long been recognised in public health that large population effects can result from fairly small individual changes. Consequently, people’s resistance may alter when they experience the extent of the changes they need to make, and perceptions like longer journey times or reduced fuel efficiency are proven unfounded. Yet, it is reported that public perceptions often limit transport and public health policy. When is this political process being oversensitive, and when do we have good enough evidence to implement a policy that could save lives and improve health?

## 7 Funding sources

The ‘Is 20 plenty for health?’ study is funded by a National Institute for Health Research (NIHR) Public Health Research (PHR) grant 15/82/12. This paper presents independent research funded by the National Institute for Health Research (NIHR). The views expressed are those of the authors and not necessarily those of the NHS, the NIHR or the Department of Health and Social Care.

## 8 Data statement

Due to the sensitive nature of the questions asked in this study, survey respondents were assured raw data would remain confidential and would not be shared.

### CRediT authorship contribution statement

**Andrew James Williams:** Conceptualization, Methodology, Formal analysis, Writing – original draft, Writing – review & editing, Visualization, Project administration, Funding acquisition. **Jillian Manner:** Investigation, Writing – review & editing. **Glenna Nightingale:** Methodology, Data curation, Project administration, Writing – review & editing. **Kieran Turner:** Conceptualization, Writing – review & editing. **Paul Kelly:** Conceptualization, Methodology, Writing – review & editing, Funding acquisition. **Graham Baker:** Conceptualization, Methodology, Writing – review & editing, Funding acquisition. **Claire Cleland:** Methodology, Writing – review & editing. **Ruth Hunter:** Conceptualization, Methodology, Writing – review & editing, Funding acquisition. **Ruth Jepson:** Conceptualization, Methodology, Writing – review & editing, Visualization, Project administration, Funding acquisition.

## Declaration of Competing Interest

The authors declare that they have no known competing financial interests or personal relationships that could have appeared to influence the work reported in this paper.
