# Supplementary material for: Public attitudes to, and perceived impacts of 20mph (32 km/h) speed limits in Edinburgh: An exploratory study using the Speed Limits Perceptions Survey (SLiPS)
Source: Transp Res Part F Traffic Psychol Behav. 2022 Jan;84:99–113. doi: 10.1016/j.trf.2021.11.022 (PMC7612163; doi:10.1016/j.trf.2021.11.022)

Appendix 1 – Speed Limits Perception Survey (SLIPS)

Adapt the survey to the specific study, location and intervention, items for adaptation enclosed in <>.

Speed Limits Perception Survey (SLIPS)

Study Information

<Insert study information, e.g. *This short survey is investigating Edinburgh residents, living, working or travelling in **Zone 6** (see map), and their perceptions of the new 20mph speed limits being implemented throughout the city. It is independent research led by the University of Edinburgh and funded by the National Institute of Health Research.*>

The survey consists of **17 questions** and should take around **10 minutes** to complete.

The survey will not be assessed or marked, so please be as honest as you can.

Please complete all questions on your own as best you can, but ask the researchers if there is anything you do not understand. You can decide to stop taking the survey at any time.

All responses will be treated confidentially and all reporting will be anonymous.

We hope to use the results to better understand how the <20mph> speed limit influences driver perceptions and behaviors. We will report the findings in scientific journals, at conferences, and on social media. We may also produce short reports to <The City of Edinburgh Council>.

Participant consent

For ethical reasons, we need to ask for your consent before you complete the survey.

By ticking this box, you agree to take the survey and give your consent for your anonymous responses to be used in the study.

☐ **I understand what I am being asked to do and agree to take part**

---

1. What is the postcode of the area of <Edinburgh> you LIVE in? ☐☐☐☐☐☐☐

Area/suburb ____________________

2. What is the postcode of the area of <Edinburgh> you WORK in? ☐☐☐☐☐☐☐

Area/suburb ____________________

Tick here if you are not working ☐

3. Which of the following statements applies to the street or road you live on / work on / drive through?

- ☐ The street/road where I live/work has <20mph> limits on all or some of it
- ☐ The street/road where I live/work does not have any <20mph> limits
- ☐ I do not drive through roads with <20mph> speed limits
- ☐ I am not sure/don't know

4. Are you aware of any (more) plans for <20mph> limits in the area where you live?

- ☐ Yes
- ☐ No
- ☐ I am not sure/don't know

5. How strongly do you agree or disagree with the following statements?
Please remember there are no right or wrong answers

Please place a tick in the appropriate columns	Strongly agree	Tend to agree	Neither agree nor disagree	Tend to disagree	Strongly disagree
<20mph> speed limits <will make/have made> people drive slower					
I understand why <Edinburgh City Council> introduced <20mph> speed limits					
<20mph> speed limits are a bad idea					
We only need <20mph> limits where safety is an issue (e.g. schools, dangerous junctions)					
We do not need <20mph> limits at night when roads are quieter					
<20mph> speed limits will take time to get used to, but eventually <20mph> will be accepted as the normal speed					

6. How much do you agree or disagree with the following statements?
'<20mph> SPEED LIMITS <WILL/HAVE> LED TO…...'

Please place a tick in the appropriate columns	Strongly agree	Tend to agree	Neither agree nor disagree	Tend to disagree	Strongly disagree
An increase in people cycling					
A decrease in the number of *severe* collisions					
An increase in people walking on the streets					
Better traffic flow (less stopping and starting)					
Decreases in the number of collisions					
More congestion					
More air pollution					
Longer journey times					
Less noise from vehicles					
Decrease in fuel efficiency					
An increase in how pleasant the area is to live or work in					
Safer streets					
More opportunities to socialise					

7. How often do you use the following transport to travel around <Edinburgh>?

Please place a tick in the appropriate columns	Every day	Several times a week	About once a week	About once a fortnight	About once a month	Less than once a month	Never
Bus, train or tram							
Motorcycle/ scooter/ moped							
Car or van							
Taxi/Uber							
Bicycle							
Walking or running							

8a. Which of the following apply to you in terms of CYCLING in Edinburgh? Tick all that apply.

- ☐ I don’t cycle and never will
- ☐ I feel safe cycling on roads with a speed limit of 20mph
- ☐ I feel safe cycling on roads with a speed limit of 30mph
- ☐ I would cycle more if there were more roads with <20mph> speed limits
- ☐ I feel unsafe cycling in <Edinburgh>
- ☐ Other road safety policies (e.g. cycle lanes) would make me cycle more

8b. If other road safety policies would make you cycle more, please indicate which ones below:

____

9. Which of the following apply to you in terms of WALKING in Edinburgh? Tick all that apply.

- ☐ I don’t walk around <Edinburgh> and never will
- ☐ I feel safe crossing roads with a speed limit of 20mph
- ☐ I feel safe crossing roads with a speed limit of 30mph
- ☐ I feel safe walking near roads with a speed limit of 20mph
- ☐ I feel safe walking near roads with a speed limit of 30mph
- ☐ I would walk more if there were more roads with <20mph> speed limits
- ☐ I feel unsafe walking in <Edinburgh>

10. Which of the following apply to you in terms of CHILDREN in Edinburgh? Tick all that apply.

- ☐ I don’t have children
- ☐ I feel children are safe near roads with a speed limit of 20mph
- ☐ I feel children are safe near roads with a speed limit of 30mph
- ☐ I feel children are safe crossing roads with a speed limit of 20mph
- ☐ I feel children are safe crossing roads with a speed limit of 30mph
- ☐ I would let children walk more if there were more roads with <20mph> speed limits
- ☐ I feel children are unsafe walking in Edinburgh due to the traffic and speed

11. Which of the following best describes your driving status and experience?

- ☐ I hold a <UK> driving license *Please state years held:* _________
- ☐ I hold a non-<UK> driving license and I drive in the UK *Please state years held:* _________
- ☐ I am a learner driver
- ☐ I have a provisional license
- ☐ I do not hold any driving license (**PLEASE GO TO Q13**)

12. How strongly do you agree or disagree with the following statements?
There are no right or wrong answers

Please place a tick in the appropriate columns	Strongly agree	Tend to agree	Neither agree nor disagree	Tend to Disagree	Strongly disagree
I use my own judgment, not speed limits, to decide on my speed on the road					
I am careful to drive at <20mph> wherever limits are					
If I think a road with <20mph> limit has no traffic I may drive faster than <20mph>					
It is just too difficult to stay at <20mph>					
I will ignore the <20mph> limits if I think I will not get caught by the police					
I get frustrated when I have to reduce my speed because someone in front of me is driving at <20mph>					
I feel pressured when I drive at <20mph> in a <20mph> zone when the driver behind me seems annoyed					
There is a high rate of road collisions caused by speeding in Edinburgh					
I tend to drive at the speed of other people on the road					
People will ignore <20mph> limits because they don't see themselves getting caught by the police					
Slowly but surely <20mph> limits are making me drive slower					
I think <20mph> speed limits will make my journeys longer					
As long as I understand the reasons for the <20mph> speed limit I will drive within the limit					

13. What is your year of birth? __ __ __ __

14. What is your gender? Male / Female / Other / Prefer not to say

15. Do you find it difficult to get around because of a permanent disability or a medical condition? Yes / No

If yes, feel free to tell us more ____

16. Which of the following below best describes your ethnic group?
Please place a tick next to the appropriate category.

**Ethnic group**	**Selection**
White	
Mixed/Multiple ethnic groups	
Asian/Asian Scottish/Asian British	
African	
Caribbean or Black	
Other ethnic group (please specify)	

17. Please write down any comments you may have on the <20mph> zones/limits:

____

____

<FINALLY

We would like to contact you in the future to invite you to take part in a short group discussion exploring your views on the <20mph> scheme. You do not have to take part in this group if you do not wish. If you are happy to be contacted to be given more information, please tick the box below and provide us with your name and address. You can decide not to take part at any time.

I am interested in finding out more about the short group discussions, and agree to be contacted in the future ☐

Name: ____
Telephone Number: ______________________________
And/or Email Address: ___________________________

If you wish to find out more about the study, please take one of our postcards.>

THANK YOU FOR TAKING PART IN THIS SURVEY!

Appendix 2 – Students who collected and extracted the survey data

Doofan	ABAA	Samuel	KERR	Alice	SCHINCO
Sophia	ABNER	Minh Duong	LE DUC	Rosa Ann	SEIDLER
Adaora	ASIKA	Elizabeth	LEHMAN	Sen	SHEN
Ijeoma	AZODO	Sarah	MACDOUGALL	Negus	SHONHIWA
Justin	BAIRD-DELHAYE	Lois	MACKAY	Laura-Beth	SPROULE
Sidra	BASHIR	Stephen	MALDEN	Jocelyn	STRAWSON
David	BELLINI	Roddy	MCDOUGALL	Xiyao	SUN
Louis	BEZANTS	Fraser	MENZIES	Yiling	TANG
Graham	BRUCE	Bethany	MILFORD	Dorothy	TERHUNE
Audrey	BUELO	Ruth	MOULSON	Suyan	TIAN
Daniel	DALLAND	Kirsten	MULLEN	Diana Herrera	VELASQUEZ
James	DUNN	Xiaoxiao	NIU	Jingchen (Estelle)	WENG
Katie	HALL	Haley	ONG	Rakhmat (Ari)	WIBOWO
Niamh	HART	Xiwu	PENG	Chloë	WILLIAMSON
Evy	HORTON	Rachel	REEL	Supei	YAN
Ying	HUANG	Nicole	ROBERTSON	Costa	YAREMENKO
Mariam	KAGALWALA	Koorosh	ROODBARAKY	Yuling	YUAN
Bridget	KEBIRUNGI	Arora	SAGAR	Minzhi	ZHOU

Appendix 3 – Data collection dates and location type

Zone and time point	Date	Location(s) type
Implementation zones 4 and 5 (16th August 2017)		
Baseline	10th August 2017 (Thursday)	Shopping Centre
	11th August 2017 (Friday)	Shopping Centre
	12th August 2017 (Saturday)	Shopping Centre
	13th August 2017 (Sunday)	Shopping Centre
6 months	9th February 2018 (Friday)	Shopping Centre
	10th February 2018 (Saturday)	Shopping Centres (x2)
	23rd February 2018 (Saturday)	Shopping Centre
12 months	9th August 2018 (Thursday)	Shopping Centres (x2)
	11th August 2018 (Saturday)	Shopping Centres (x2)
Implementation zone 6 (5th March 2018)		
Baseline	9th February 2018 (Friday)	Shopping Centre & Sports Centre
	10th February 2018 (Saturday)	Shopping Centre & Sports Centre
6 months	9th August 2018 (Thursday)	Shopping Centre & Sports Centre
	11th August 2018 (Saturday)	Shopping Centre
	30th August 2018 (Thursday)	Hospital
	1st September 2018 (Saturday)	Hospital
	6th September 2018 (Thursday)	Hospital
12 months	15th January 2019 (Tuesday)	Hospital
	17th January 2019 (Thursday)	Hospital
	19th January 2019 (Saturday)	Hospital
	22nd January 2019 (Tuesday)	Hospital
	24th January 2019 (Thursday)	Hospital

Appendix 4 – Participant perceptions at each time point and changes

**Survey question**	Survey responses	Baseline (**A**)	6 months (**B**)	1 year (**C**)	6 month change (**B-A**)	1 year change (**C-A**)
General attitudes to the road						
I use my own judgment, not speed limits, to decide on my speed on the road	Strongly agree	14.34% (146)	13.52% (81)	8.96% (57)	-0.82	-5.38
	Tend to agree	18.66% (190)	20.37% (122)	21.23% (135)	1.71	2.57
	Neither agree nor disagree	15.82% (161)	11.85% (71)	11.48% (73)	-3.97	-4.34
	Tend to disagree	26.03% (265)	25.88% (155)	26.57% (169)	-0.15	0.54
	Strongly disagree	25.15% (256)	28.38% (170)	31.76% (202)	3.23	6.61
I tend to drive at the speed of other people on the road	Strongly agree	6.39% (65)	8.51% (51)	6.45% (41)	2.12	0.06
	Tend to agree	35.66% (363)	34.56% (207)	34.91% (222)	-1.10	-0.75
	Neither agree nor disagree	19.35% (197)	18.36% (110)	21.07% (134)	-0.99	1.72
	Tend to disagree	25.44% (259)	23.37% (140)	26.1% (166)	-2.07	0.66
	Strongly disagree	13.16% (134)	15.19% (91)	11.48% (73)	2.03	-1.68
There is a high rate of road collisions caused by speeding in Edinburgh	Strongly agree	7.66% (78)	6.68% (40)	8.02% (51)	-0.98	0.36
	Tend to agree	14.44% (147)	13.36% (80)	15.57% (99)	-1.08	1.13
	Neither agree nor disagree	43.91% (447)	48.91% (293)	50.16% (319)	5.00	6.25
	Tend to disagree	23.18% (236)	21.7% (130)	19.03% (121)	-1.48	-4.15
	Strongly disagree	10.81% (110)	9.35% (56)	7.23% (46)	-1.46	-3.58
**I feel safe walking near roads with a speed limit of…**	Always unsafe	2.16% (22)	2.17% (13)	2.52% (16)	0.01	0.36
	<20mph	24.95% (254)	22.54% (135)	18.71% (119)	-2.41	-6.24
	20mph	7.66% (78)	10.52% (63)	12.58% (80)	2.86	4.92
	30mph	65.23% (664)	64.77% (388)	66.19% (421)	-0.46	0.96
I feel safe crossing roads with a speed limit of…	<20mph	15.91% (162)	15.53% (93)	14.15% (90)	-0.38	-1.76
	20mph	12.38% (126)	14.86% (89)	15.41% (98)	2.48	3.03
	30mph	71.71% (730)	69.62% (417)	70.44% (448)	-2.09	-1.27
**I feel safe cycling on roads with a speed limit of…**	Always unsafe	23.08% (235)	18.53% (111)	19.97% (127)	-4.55	-3.11
	<20mph	54.42% (554)	57.43% (344)	55.66% (354)	3.01	1.24
	20mph	6.78% (69)	7.68% (46)	10.69% (68)	0.90	3.91
	30mph	15.72% (160)	16.36% (98)	13.68% (87)	0.64	-2.04
**I feel children are safe near roads with a speed limit of…**	Always unsafe	3.63% (37)	5.01% (30)	7.86% (50)	1.38	4.23
	<20mph	57.07% (581)	47.25% (283)	45.28% (288)	-9.82	-11.79
	20mph	15.82% (161)	23.71% (142)	25% (159)	7.89	9.18
	30mph	23.48% (239)	24.04% (144)	21.86% (139)	0.56	-1.62
**I feel children are safe crossing roads with a speed limit of…**	<20mph	66.31% (675)	61.27% (367)	61.95% (394)	-5.04	-4.36
	20mph	11.39% (116)	17.53% (105)	18.87% (120)	6.14	7.48
	30mph	22.3% (227)	21.2% (127)	19.18% (122)	-1.10	-3.12

**Survey question**	Survey responses	Baseline (**A**)	6 months (**B**)	1 year (**C**)	6 month change (**B-A**)	1 year change (**C-A**)
Attitudes to 20mph limits						
**I am careful to drive at 20mph wherever limits are**	Strongly agree	25.64% (261)	31.55% (189)	28.77% (183)	5.91	3.13
	Tend to agree	34.77% (354)	33.72% (202)	38.21% (243)	-1.05	3.44
	Neither agree nor disagree	15.23% (155)	10.52% (63)	12.89% (82)	-4.71	-2.34
	Tend to disagree	18.86% (192)	16.53% (99)	15.88% (101)	-2.33	-2.98
	Strongly disagree	5.5% (56)	7.68% (46)	4.25% (27)	2.18	-1.25
**I understand why Edinburgh City Council introduced 20mph speed limits**	Strongly agree	20.83% (212)	28.71% (172)	27.83% (177)	7.88	7.00
	Tend to agree	31.14% (317)	32.55% (195)	38.52% (245)	1.41	7.38
	Neither agree nor disagree	11.59% (118)	9.52% (57)	10.22% (65)	-2.07	-1.37
	Tend to disagree	16.7% (170)	13.69% (82)	13.68% (87)	-3.01	-3.02
	Strongly disagree	19.74% (201)	15.53% (93)	9.75% (62)	-4.21	-9.99
As long as I understand the reasons for the 20mph speed limit I will drive within the limit	Strongly agree	22.79% (232)	26.54% (159)	21.38% (136)	3.75	-1.41
	Tend to agree	29.76% (303)	31.05% (186)	31.13% (198)	1.29	1.37
	Neither agree nor disagree	28.49% (290)	23.21% (139)	30.35% (193)	-5.28	1.86
	Tend to disagree	11.79% (120)	11.85% (71)	10.38% (66)	0.06	-1.41
	Strongly disagree	7.17% (73)	7.35% (44)	6.76% (43)	0.18	-0.41
**20mph speed limits will take time to get used to, but eventually 20mph will be accepted as the normal speed**	Strongly agree	10.12% (103)	12.02% (72)	11.95% (76)	1.90	1.83
	Tend to agree	23.58% (240)	28.05% (168)	28.30 (180)	4.47	4.72
	Neither agree nor disagree	12.97% (132)	12.19% (73)	14.47% (92)	-0.78	1.50
	Tend to disagree	28.98% (295)	25.71% (154)	27.36% (174)	-3.27	-1.62
	Strongly disagree	24.36% (248)	22.04% (132)	17.92% (114)	-2.32	-6.44
**I think 20mph speed limits will make my journeys longer**	Strongly agree	45.48% (463)	37.56% (225)	35.06% (223)	-7.92	-10.42
	Tend to agree	28.88% (294)	32.55% (195)	30.66% (195)	3.67	1.78
	Neither agree nor disagree	12.08% (123)	14.02% (84)	16.35% (104)	1.94	4.27
	Tend to disagree	10.22% (104)	11.35% (68)	12.89% (82)	1.13	2.67
	Strongly disagree	3.34% (34)	4.51% (27)	5.03% (32)	1.17	1.69
**It is just too difficult to stay at 20mph**	Strongly agree	30.45% (310)	25.71% (154)	24.06% (153)	-4.74	-6.39
	Tend to agree	34.18% (348)	32.05% (192)	33.81% (215)	-2.13	-0.37
	Neither agree nor disagree	15.03% (153)	15.36% (92)	14.62% (93)	0.33	-0.41
	Tend to disagree	11.2% (114)	14.36% (86)	14.94% (95)	3.16	3.74
	Strongly disagree	9.14% (93)	12.52% (75)	12.58% (80)	3.38	3.44
**20mph speed limits are a bad idea**	Strongly agree	27.5% (280)	23.04% (138)	18.4% (117)	-4.46	-9.10
	Tend to agree	16.4% (167)	14.19% (85)	15.88% (101)	-2.21	-0.52
	Neither agree nor disagree	15.91% (162)	15.36% (92)	18.71% (119)	-0.55	2.80
	Tend to disagree	20.24% (206)	23.54% (141)	20.91% (133)	3.30	0.67
	Strongly disagree	19.94% (203)	23.87% (143)	26.1% (166)	3.93	6.16

**Survey question**	Survey responses	Baseline (**A**)	6 months (**B**)	1 year (**C**)	6 month change (**B-A**)	1 year change (**C-A**)
Attitudes to 20mph limits						
We do not need 20mph limits at night when roads are quieter*	Strongly agree	33.33% (106)	39.23% (235)	31.13% (198)	5.90	-2.20
	Tend to agree	21.07% (67)	19.03% (114)	23.74% (151)	-2.04	2.67
	Neither agree nor disagree	11.32% (36)	10.68% (64)	13.05% (83)	-0.64	1.73
	Tend to disagree	18.87% (60)	15.86% (95)	17.3% (110)	-3.01	-1.57
	Strongly disagree	15.41% (49)	15.19% (91)	14.78% (94)	-0.22	-0.63
We only need 20mph limits where safety is an issue (e.g. schools, dangerous junctions)*	Strongly agree	43.4% (138)	46.41% (278)	44.03% (280)	3.01	0.63
	Tend to agree	23.58% (75)	22.37% (134)	23.58% (150)	-1.21	0.00
	Neither agree nor disagree	8.49% (27)	7.35% (44)	5.19% (33)	-1.14	-3.30
	Tend to disagree	15.09% (48)	12.35% (74)	15.57% (99)	-2.74	0.48
	Strongly disagree	9.43% (30)	11.52% (69)	11.64% (74)	2.09	2.21
People will ignore 20mph limits because they don't see themselves getting caught by the police	Strongly agree	28.98% (295)	25.04% (150)	28.46% (181)	-3.94	-0.52
	Tend to agree	44.3% (451)	47.58% (285)	44.81% (285)	3.28	0.51
	Neither agree nor disagree	15.42% (157)	17.03% (102)	16.19% (103)	1.61	0.77
	Tend to disagree	8.74% (89)	6.68% (40)	7.86% (50)	-2.06	-0.88
	Strongly disagree	2.55% (26)	3.67% (22)	2.67% (17)	1.12	0.12
I will ignore the 20mph limits if I think I will not get caught by the police	Strongly agree	8.84% (90)	9.68% (58)	7.23% (46)	0.84	-1.61
	Tend to agree	17.68% (180)	15.19% (91)	15.72% (100)	-2.49	-1.96
	Neither agree nor disagree	18.07% (184)	16.36% (98)	16.35% (104)	-1.71	-1.72
	Tend to disagree	22.69% (231)	24.54% (147)	25% (159)	1.85	2.31
	Strongly disagree	32.71% (333)	34.22% (205)	35.69% (227)	1.51	2.98
If I think a road with 20mph limit has no traffic, I may drive faster than 20mph	Strongly agree	14.34% (146)	15.69% (94)	13.84% (88)	1.35	-0.50
	Tend to agree	37.82% (385)	34.72% (208)	33.65% (214)	-3.10	-4.17
	Neither agree nor disagree	12.67% (129)	11.52% (69)	15.25% (97)	-1.15	2.58
	Tend to disagree	19.55% (199)	17.03% (102)	19.97% (127)	-2.52	0.42
	Strongly disagree	15.62% (159)	21.04% (126)	17.3% (110)	5.42	1.68
I get frustrated when I have to reduce my speed because someone in front of me is driving at 20mph	Strongly agree	15.03% (153)	16.03% (96)	14.15% (90)	1.00	-0.88
	Tend to agree	22.69% (231)	19.37% (116)	18.24% (116)	-3.32	-4.45
	Neither agree nor disagree	16.6% (169)	15.36% (92)	17.77% (113)	-1.24	1.17
	Tend to disagree	21.12% (215)	23.21% (139)	22.64% (144)	2.09	1.52
	Strongly disagree	24.56% (250)	26.04% (156)	27.2% (173)	1.48	2.64
I feel pressured when I drive at 20mph in a 20mph zone when the driver behind me seems annoyed	Strongly agree	32.81% (334)	29.55% (177)	31.6% (201)	-3.26	-1.21
	Tend to agree	32.12% (327)	28.55% (171)	33.02% (210)	-3.57	0.90
	Neither agree nor disagree	12.18% (124)	15.03% (90)	10.85% (69)	2.85	-1.33
	Tend to disagree	11% (112)	11.85% (71)	11.48% (73)	0.85	0.48

	Strongly disagree	11.89% (121)	15.03% (90)	13.05% (83)	3.14	1.16

**Survey question**	Survey responses	Baseline (**A**)	6 months (**B**)	1 year (**C**)	6 month change (**B-A**)	1 year change (**C-A**)
Attitudes to 20mph limits						
I would walk more if there were more roads with 20mph speed limits	No	93.71% (954)	92.65% (555)	91.67% (583)	-1.06	-2.04
	Yes	6.29% (64)	7.35% (44)	8.33% (53)	1.06	2.04
I would cycle more if there were more roads with 20mph speed limits	No	92.73% (944)	92.99% (557)	91.67% (583)	0.26	-1.06
	Yes	7.27% (74)	7.01% (42)	8.33% (53)	-0.26	1.06
Perceived impacts of 20mph limits						
**20mph speed limits will/have made people drive slower**	Strongly agree	13.26% (135)	16.53% (99)	12.11% (77)	3.27	-1.15
	Tend to agree	33.01% (336)	36.23% (217)	42.3% (269)	3.22	9.29
	Neither agree nor disagree	9.92% (101)	12.35% (74)	10.53% (67)	2.43	0.61
	Tend to disagree	26.82% (273)	21.87% (131)	22.8% (145)	-4.95	-4.02
	Strongly disagree	16.99% (173)	13.02% (78)	12.26% (78)	-3.97	-4.73
Slowly but surely 20mph limits are making me drive slower	Strongly agree	8.45% (86)	10.35% (62)	9.59% (61)	1.90	1.14
	Tend to agree	27.6% (281)	30.22% (181)	31.76% (202)	2.62	4.16
	Neither agree nor disagree	28.88% (294)	23.54% (141)	28.77% (183)	-5.34	-0.11
	Tend to disagree	22.3% (227)	22.37% (134)	19.34% (123)	0.07	-2.96
	Strongly disagree	12.77% (130)	13.52% (81)	10.53% (67)	0.75	-2.24
**20mph speed limits will/have led to longer journey times**	Strongly agree	47.35% (482)	40.23% (241)	35.85% (228)	-7.12	-11.50
	Tend to agree	31.63% (322)	32.55% (195)	34.59% (220)	0.92	2.96
	Neither agree nor disagree	9.43% (96)	13.52% (81)	14.15% (90)	4.09	4.72
	Tend to disagree	7.27% (74)	9.68% (58)	11.32% (72)	2.41	4.05
	Strongly disagree	4.32% (44)	4.01% (24)	4.09% (26)	-0.31	-0.23
**20mph speed limits will/have led to better traffic flow (less stopping and starting)**	Strongly agree	4.91% (50)	3.51% (21)	3.14% (20)	-1.40	-1.77
	Tend to agree	11% (112)	9.02% (54)	13.52% (86)	-1.98	2.52
	Neither agree nor disagree	12.57% (128)	21.2% (127)	19.03% (121)	8.63	6.46
	Tend to disagree	29.27% (298)	29.55% (177)	32.55% (207)	0.28	3.28
	Strongly disagree	42.24% (430)	36.73% (220)	31.76% (202)	-5.51	-10.48
**20mph speed limits will/have led to more congestion**	Strongly agree	35.27% (359)	30.38% (182)	25.63% (163)	-4.89	-9.64
	Tend to agree	28.88% (294)	26.21% (157)	29.25% (186)	-2.67	0.37
	Neither agree nor disagree	17.49% (178)	24.21% (145)	23.58% (150)	6.72	6.09
	Tend to disagree	11.69% (119)	13.19% (79)	16.67% (106)	1.50	4.98
	Strongly disagree	6.68% (68)	6.01% (36)	4.87% (31)	-0.67	-1.81

**Survey question**	Survey responses	Baseline (**A**)	6 months (**B**)	1 year (**C**)	6 month change (**B-A**)	1 year change (**C-A**)
Perceived impacts of 20mph limits						
**20mph speed limits will/have led to more air pollution**	Strongly agree	38.41% (391)	31.55% (189)	22.48% (143)	-6.86	-15.93
	Tend to agree	25.25% (257)	22.37% (134)	25.79% (164)	-2.88	0.54
	Neither agree nor disagree	18.66% (190)	31.22% (187)	31.13% (198)	12.56	12.47
	Tend to disagree	11.39% (116)	9.02% (54)	15.25% (97)	-2.37	3.86
	Strongly disagree	6.29% (64)	5.84% (35)	5.35% (34)	-0.45	-0.94
**20mph speed limits will/have led to less noise from vehicles***	Strongly agree	6.29% (20)	3.51% (21)	4.56% (29)	-2.78	-1.73
	Tend to agree	23.27% (74)	12.69% (76)	17.3% (110)	-10.58	-5.97
	Neither agree nor disagree	31.76% (101)	39.9% (239)	38.84% (247)	8.14	7.08
	Tend to disagree	23.9% (76)	25.04% (150)	26.42% (168)	1.14	2.52
	Strongly disagree	14.78% (47)	18.86% (113)	12.89% (82)	4.08	-1.89
**20mph speed limits will/have led to decrease in fuel efficiency**	Strongly agree	31.93% (325)	26.21% (157)	20.75% (132)	-5.72	-11.18
	Tend to agree	21.91% (223)	19.03% (114)	20.75% (132)	-2.88	-1.16
	Neither agree nor disagree	26.42% (269)	36.23% (217)	36.64% (233)	9.81	10.22
	Tend to disagree	11% (112)	10.18% (61)	13.05% (83)	-0.82	2.05
	Strongly disagree	8.74% (89)	8.35% (50)	8.81% (56)	-0.39	0.07
**20mph speed limits will/have led to decreases in the number of collisions**	Strongly agree	9.04% (92)	5.34% (32)	3.77% (24)	-3.70	-5.27
	Tend to agree	25.83% (263)	19.2% (115)	20.28% (129)	-6.63	-5.55
	Neither agree nor disagree	18.86% (192)	41.24% (247)	46.54% (296)	22.38	27.68
	Tend to disagree	27.31% (278)	19.87% (119)	20.28% (129)	-7.44	-7.03
	Strongly disagree	18.96% (193)	14.36% (86)	9.12% (58)	-4.60	-9.84
**20mph speed limits will/have led to a decrease in the number of severe collisions**	Strongly agree	13.95% (142)	7.85% (47)	6.29% (40)	-6.10	-7.66
	Tend to agree	33.3% (339)	21.87% (131)	25.31% (161)	-11.43	-7.99
	Neither agree nor disagree	16.9% (172)	40.4% (242)	42.77% (272)	23.50	25.87
	Tend to disagree	23.77% (242)	17.36% (104)	16.98% (108)	-6.41	-6.79
	Strongly disagree	12.08% (123)	12.52% (75)	8.65% (55)	0.44	-3.43
**20mph speed limits will/have led to safer streets***	Strongly agree	20.13% (64)	13.36% (80)	14.47% (92)	-6.77	-5.66
	Tend to agree	32.08% (102)	26.88% (161)	29.87% (190)	-5.20	-2.21
	Neither agree nor disagree	20.75% (66)	26.71% (160)	26.89% (171)	5.96	6.14
	Tend to disagree	18.87% (60)	19.03% (114)	19.65% (125)	0.16	0.78
	Strongly disagree	8.18% (26)	14.02% (84)	9.12% (58)	5.84	0.94
**20mph speed limits will/have led to an increase in how pleasant the area is to live or work in***	Strongly agree	15.72% (50)	8.35% (50)	10.38% (66)	-7.37	-5.34
	Tend to agree	16.98% (54)	17.03% (102)	17.45% (111)	0.05	0.47
	Neither agree nor disagree	39.31% (125)	39.73% (238)	36.64% (233)	0.42	-2.67
	Tend to disagree	17.61% (56)	16.53% (99)	21.23% (135)	-1.08	3.62

**Survey question**	Survey responses	Baseline (**A**)	6 months (**B**)	1 year (**C**)	6 month change (**B-A**)	1 year change (**C-A**)
	Strongly disagree	10.38% (33)	18.36% (110)	14.31% (91)	7.98	3.93
Perceived impacts of 20mph limits						
20mph speed limits will/have led to more opportunities to socialise*	Strongly agree	3.46% (11)	2.67% (16)	2.99% (19)	-0.79	-0.47
	Tend to agree	5.03% (16)	4.01% (24)	3.3% (21)	-1.02	-1.73
	Neither agree nor disagree	34.59% (110)	38.73% (232)	38.68% (246)	4.14	4.09
	Tend to disagree	27.36% (87)	21.54% (129)	21.7% (138)	-5.82	-5.66
	Strongly disagree	29.56% (94)	33.06% (198)	33.33% (212)	3.50	3.77
**20mph speed limits will/have led to an increase in people walking on the streets**	Strongly agree	5.99% (61)	4.01% (24)	2.83% (18)	-1.98	-3.16
	Tend to agree	14.73% (150)	11.19% (67)	11.01% (70)	-3.54	-3.72
	Neither agree nor disagree	26.62% (271)	41.24% (247)	42.92% (273)	14.62	16.30
	Tend to disagree	34.28% (349)	26.04% (156)	29.87% (190)	-8.24	-4.41
	Strongly disagree	18.37% (187)	17.53% (105)	13.36% (85)	-0.84	-5.01
**20mph speed limits will/have led to an increase in people cycling**	Strongly agree	6.68% (68)	5.51% (33)	5.03% (32)	-1.17	-1.65
	Tend to agree	14.54% (148)	12.52% (75)	13.68% (87)	-2.02	-0.86
	Neither agree nor disagree	30.65% (312)	47.41% (284)	44.65% (284)	16.76	14.00
	Tend to disagree	28.09% (286)	21.54% (129)	24.69% (157)	-6.55	-3.40
	Strongly disagree	20.04% (204)	13.02% (78)	11.95% (76)	-7.02	-8.09

Variables in bold vary statistically significantly across time points
*These questions were not asked until after baseline data had been collected in Zones 5 and 5, so there are 700 fewer observations at baseline.

Appendix 5 – 20mph limit perception factors: characteristics and description

	Factor i	Factor ii	Factor iii	Factor iv	Factor v	Uniqueness
**General attitudes to the road**						
I use my own judgment, not speed limits, to decide on my speed on the road			0.5103			0.7464
I tend to drive at the speed of other people on the road			0.6565			0.6238
There is a high rate of road collisions caused by speeding in Edinburgh	0.4907					0.5830
I feel safe walking near roads with a speed limit of…					0.7732	0.3638
I feel safe crossing roads with a speed limit of…					0.7733	0.3860
I feel safe cycling on roads with a speed limit of…						0.9143
I feel children are safe near roads with a speed limit of…				0.9007		0.1839
I feel children are safe crossing roads with a speed limit of…				0.8925		0.1609
**Attitudes to 20mph limits**						
I am careful to drive at 20mph wherever limits are			-0.6384			0.4812
I understand why Edinburgh City Council introduced 20mph speed limits	0.5445					0.4702
As long as I understand the reasons for the 20mph speed limit I will drive within the limit	0.4281		-0.4019			0.5623
20mph speed limits will take time to get used to, but eventually 20mph will be accepted as the normal speed	0.6854					0.3655
I think 20mph speed limits will make my journeys longer		0.6141				0.4457
It is just too difficult to stay at 20mph		0.3237	0.4407			0.4367
20mph speed limits are a bad idea	-0.3206	0.3950				0.5210
People will ignore 20mph limits because they don't see themselves getting caught by the police						0.9593
I will ignore the 20mph limits if I think I will not get caught by the police			0.8498			0.3114
If I think a road with 20mph limit has no traffic, I may drive faster than 20mph			0.7816			0.4105
I get frustrated when I have to reduce my speed because someone in front of me is driving at 20mph			0.7080			0.3562
I feel pressured when I drive at 20mph in a 20mph zone when the driver behind me seems annoyed						0.8433
I would walk more if there were more roads with 20mph speed limits	-0.5738					0.5127
I would cycle more if there were more roads with 20mph speed limits	-0.6946					0.4229
**Perceived impacts of 20mph limits**						
20mph speed limits will/have made people drive slower	0.7331					0.4896

Slowly but surely 20mph limits are making me drive slower	0.6266					0.5166
20mph speed limits will/have led to longer journey times		0.9078				0.2015
20mph speed limits will/have led to better traffic flow (less stopping and starting)	0.6411					0.3198
20mph speed limits will/have led to more congestion		0.8526				0.2612
20mph speed limits will/have led to more air pollution		0.8585				0.2810
20mph speed limits will/have led to decrease in fuel efficiency		0.6080				0.6321
20mph speed limits will/have led to decreases in the number of collisions	0.7753					0.3323
20mph speed limits will/have led a decrease in the number of severe collisions	0.8147					0.3471
20mph speed limits will/have led to a n increase in people walking on the streets	0.6621					0.6565
20mph speed limits will/have led to an increase in people cycling	0.7426					0.5436
**Descriptive title**	*Detraction and resistance*	*Support*	*Rule following*	*Child safety*	*Walking safety*	

Factor loadings less than ±0.3 have been left blank.

Appendix 6 – Polychoric correlation matrix

General attitudes to the road	A	B	C	D	E	F	G
A. I use my own judgment, not speed limits, to decide on my speed on the road	1						
B. I tend to drive at the speed of other people on the road	0.390	1					
C. There is a high rate of road collisions caused by speeding in Edinburgh	-0.119	-0.138	1				
D. I feel safe walking near roads with a speed limit of…	0.025	-0.087	0.206	1			
E. I feel safe crossing roads with a speed limit of…	-0.076	-0.094	0.213	0.671	1		
F. I feel safe cycling on roads with a speed limit of…	-0.035	-0.078	0.107	0.014	0.147	1	
G. I feel children are safe near roads with a speed limit of…	-0.033	-0.022	0.206	0.243	0.213	0.140	1
H. I feel children are safe crossing roads with a speed limit of…	-0.014	-0.021	0.252	0.362	0.207	0.076	0.872
**Attitudes to 20mph limits**							
I. I am careful to drive at 20mph wherever limits are	-0.286	-0.435	0.359	0.049	0.122	0.059	0.112
J. I understand why Edinburgh City Council introduced 20mph speed limits	-0.228	-0.207	0.442	0.066	0.100	-0.038	0.069
K. As long as I understand the reasons for the 20mph speed limit I will drive within the limit	-0.196	-0.259	0.417	0.067	0.103	0.044	0.091
L. 20mph speed limits will take time to get used to, but eventually 20mph will be accepted as the normal speed	-0.221	-0.183	0.465	0.081	0.147	-0.007	0.055
M. I think 20mph speed limits will make my journeys longer	0.187	0.173	-0.325	-0.082	-0.100	0.047	-0.134
N. It is just too difficult to stay at 20mph	0.283	0.322	-0.352	-0.056	-0.126	0.012	-0.101
O. 20mph speed limits are a bad idea	0.196	0.189	-0.335	-0.011	-0.094	-0.004	-0.056
P. People will ignore 20mph limits because they don't see themselves getting caught by the police	-0.011	0.050	0.150	0.029	-0.045	0.051	0.069
Q. I will ignore the 20mph limits if I think I will not get caught by the police	0.415	0.485	-0.279	-0.055	-0.146	-0.083	-0.060
R. If I think a road with 20mph limit has no traffic, I may drive faster than 20mph	0.377	0.478	-0.292	-0.074	-0.135	-0.051	-0.070
S. I get frustrated when I have to reduce my speed because someone in front of me is driving at 20mph	0.387	0.443	-0.365	-0.045	-0.095	-0.033	-0.055
T. I feel pressured when I drive at 20mph in a 20mph zone when the driver behind me seems annoyed	0.035	0.138	-0.148	-0.125	-0.112	0.088	-0.042
U. I would walk more if there were more roads with 20mph speed limits	0.141	0.145	-0.464	-0.176	-0.302	0.073	0.002
V. I would cycle more if there were more roads with 20mph speed limits	0.096	0.089	-0.257	0.029	-0.098	0.311	-0.006
**Perceived impacts of 20mph limits**							
W. 20mph speed limits will/have made people drive slower	-0.146	-0.085	0.377	0.008	0.073	-0.069	-0.034
X. Slowly but surely 20mph limits are making me drive slower	-0.204	-0.227	0.448	0.076	0.121	-0.002	0.024
Y. 20mph speed limits will/have led to longer journey times	0.204	0.170	-0.269	-0.052	-0.064	0.053	-0.077
Z. 20mph speed limits will/have led to better traffic flow (less stopping and starting)	-0.135	-0.129	0.448	0.100	0.179	-0.101	0.114
AA. 20mph speed limits will/have led to more congestion	0.172	0.104	-0.239	0.022	-0.030	0.076	-0.084
BB. 20mph speed limits will/have led to more air pollution	0.186	0.139	-0.226	-0.009	-0.054	0.095	-0.047
CC. 20mph speed limits will/have led to decrease in fuel efficiency	0.149	0.145	-0.286	-0.107	-0.125	0.013	-0.070
DD. 20mph speed limits will/have led to decreases in the number of collisions	-0.199	-0.135	0.474	0.072	0.130	-0.035	0.059
EE. 20mph speed limits will/have led a decrease in the number of severe collisions	-0.167	-0.158	0.419	-0.005	0.077	-0.048	0.040
FF. 20mph speed limits will/have led to a n increase in people walking on the streets	-0.021	-0.019	0.240	0.115	0.196	-0.068	0.056

GG. 20mph speed limits will/have led to an increase in people cycling	-0.073	-0.081	0.331	0.023	0.132	-0.119	0.040

**General attitudes to the road**	**H**	**I**	**J**	**K**	**L**	**M**	**N**
A. I use my own judgment, not speed limits, to decide on my speed on the road							
B. I tend to drive at the speed of other people on the road							
C. There is a high rate of road collisions caused by speeding in Edinburgh							
D. I feel safe walking near roads with a speed limit of…							
E. I feel safe crossing roads with a speed limit of…							
F. I feel safe cycling on roads with a speed limit of…							
G. I feel children are safe near roads with a speed limit of…							
H. I feel children are safe crossing roads with a speed limit of…	1						
**Attitudes to 20mph limits**							
I. I am careful to drive at 20mph wherever limits are	0.144	1					
J. I understand why Edinburgh City Council introduced 20mph speed limits	0.111	0.373	1				
K. As long as I understand the reasons for the 20mph speed limit I will drive within the limit	0.089	0.530	0.408	1			
L. 20mph speed limits will take time to get used to, but eventually 20mph will be accepted as the normal speed	0.091	0.407	0.605	0.484	1		
M. I think 20mph speed limits will make my journeys longer	-0.112	-0.255	-0.482	-0.297	-0.451	1	
N. It is just too difficult to stay at 20mph	-0.089	-0.382	-0.514	-0.386	-0.495	0.547	1
O. 20mph speed limits are a bad idea	-0.085	-0.313	-0.533	-0.359	-0.519	0.489	0.497
P. People will ignore 20mph limits because they don't see themselves getting caught by the police	0.090	0.019	-0.004	0.048	-0.031	0.092	0.127
Q. I will ignore the 20mph limits if I think I will not get caught by the police	-0.034	-0.587	-0.313	-0.423	-0.358	0.306	0.510
R. If I think a road with 20mph limit has no traffic, I may drive faster than 20mph	-0.046	-0.525	-0.280	-0.358	-0.329	0.285	0.532
S. I get frustrated when I have to reduce my speed because someone in front of me is driving at 20mph	-0.062	-0.527	-0.417	-0.465	-0.436	0.457	0.616
T. I feel pressured when I drive at 20mph in a 20mph zone when the driver behind me seems annoyed	-0.022	-0.089	-0.215	-0.144	-0.221	0.308	0.411
U. I would walk more if there were more roads with 20mph speed limits	-0.061	-0.334	-0.397	-0.315	-0.489	0.363	0.307
V. I would cycle more if there were more roads with 20mph speed limits	-0.100	-0.273	-0.556	-0.224	-0.488	0.383	0.458
**Perceived impacts of 20mph limits**							
W. 20mph speed limits will/have made people drive slower	0.039	0.335	0.500	0.383	0.595	-0.328	-0.388
X. Slowly but surely 20mph limits are making me drive slower	0.075	0.452	0.406	0.572	0.599	-0.264	-0.319
Y. 20mph speed limits will/have led to longer journey times	-0.115	-0.245	-0.428	-0.264	-0.442	0.686	0.458
Z. 20mph speed limits will/have led to better traffic flow (less stopping and starting)	0.161	0.295	0.610	0.395	0.611	-0.571	-0.533
AA. 20mph speed limits will/have led to more congestion	-0.079	-0.206	-0.417	-0.235	-0.419	0.581	0.396
BB. 20mph speed limits will/have led to more air pollution	-0.066	-0.218	-0.413	-0.246	-0.423	0.536	0.431
CC. 20mph speed limits will/have led to decrease in fuel efficiency	-0.080	-0.225	-0.296	-0.177	-0.313	0.373	0.321
DD. 20mph speed limits will/have led to decreases in the number of collisions	0.102	0.283	0.589	0.427	0.662	-0.449	-0.481
EE. 20mph speed limits will/have led a decrease in the number of severe collisions	0.088	0.326	0.577	0.410	0.624	-0.428	-0.431
FF. 20mph speed limits will/have led to a n increase in people walking on the streets	0.115	0.120	0.290	0.250	0.393	-0.215	-0.257

GG. 20mph speed limits will/have led to an increase in people cycling	0.096	0.216	0.371	0.293	0.464	-0.313	-0.313

**General attitudes to the road**	**O**	**P**	**Q**	**R**	**S**	**T**	**U**
A. I use my own judgment, not speed limits, to decide on my speed on the road							
B. I tend to drive at the speed of other people on the road							
C. There is a high rate of road collisions caused by speeding in Edinburgh							
D. I feel safe walking near roads with a speed limit of…							
E. I feel safe crossing roads with a speed limit of…							
F. I feel safe cycling on roads with a speed limit of…							
G. I feel children are safe near roads with a speed limit of…							
H. I feel children are safe crossing roads with a speed limit of…							
**Attitudes to 20mph limits**							
I. I am careful to drive at 20mph wherever limits are							
J. I understand why Edinburgh City Council introduced 20mph speed limits							
K. As long as I understand the reasons for the 20mph speed limit I will drive within the limit							
L. 20mph speed limits will take time to get used to, but eventually 20mph will be accepted as the normal speed							
M. I think 20mph speed limits will make my journeys longer							
N. It is just too difficult to stay at 20mph							
O. 20mph speed limits are a bad idea	1						
P. People will ignore 20mph limits because they don't see themselves getting caught by the police	0.029	1					
Q. I will ignore the 20mph limits if I think I will not get caught by the police	0.315	0.152	1				
R. If I think a road with 20mph limit has no traffic, I may drive faster than 20mph	0.247	0.045	0.695	1			
S. I get frustrated when I have to reduce my speed because someone in front of me is driving at 20mph	0.417	0.060	0.661	0.598	1		
T. I feel pressured when I drive at 20mph in a 20mph zone when the driver behind me seems annoyed	0.192	0.202	0.149	0.164	0.344	1	
U. I would walk more if there were more roads with 20mph speed limits	0.445	-0.018	0.181	0.237	0.315	0.242	1
V. I would cycle more if there were more roads with 20mph speed limits	0.517	0.057	0.236	0.204	0.312	0.178	0.590
**Perceived impacts of 20mph limits**							
W. 20mph speed limits will/have made people drive slower	-0.367	-0.079	-0.266	-0.215	-0.312	-0.238	-0.409
X. Slowly but surely 20mph limits are making me drive slower	-0.356	0.008	-0.360	-0.306	-0.413	-0.101	-0.374
Y. 20mph speed limits will/have led to longer journey times	0.543	0.045	0.237	0.235	0.363	0.254	0.323
Z. 20mph speed limits will/have led to better traffic flow (less stopping and starting)	-0.542	-0.059	-0.275	-0.255	-0.391	-0.251	-0.476
AA. 20mph speed limits will/have led to more congestion	0.470	0.036	0.170	0.176	0.278	0.193	0.359
BB. 20mph speed limits will/have led to more air pollution	0.500	0.041	0.168	0.192	0.299	0.204	0.369
CC. 20mph speed limits will/have led to decrease in fuel efficiency	0.397	0.026	0.138	0.201	0.226	0.115	0.264
DD. 20mph speed limits will/have led to decreases in the number of collisions	-0.495	-0.063	-0.286	-0.240	-0.393	-0.217	-0.486
EE. 20mph speed limits will/have led a decrease in the number of severe collisions	-0.472	-0.050	-0.309	-0.240	-0.383	-0.216	-0.440
FF. 20mph speed limits will/have led to a n increase in people walking on the streets	-0.247	0.011	-0.126	-0.125	-0.169	-0.162	-0.344
GG. 20mph speed limits will/have led to an increase in people cycling	-0.313	-0.032	-0.160	-0.169	-0.225	-0.136	-0.403

**General attitudes to the road**	**V**	**W**	**X**	**Y**	**Z**	**AA**	**BB**
A. I use my own judgment, not speed limits, to decide on my speed on the road							
B. I tend to drive at the speed of other people on the road							
C. There is a high rate of road collisions caused by speeding in Edinburgh							
D. I feel safe walking near roads with a speed limit of…							
E. I feel safe crossing roads with a speed limit of…							
F. I feel safe cycling on roads with a speed limit of…							
G. I feel children are safe near roads with a speed limit of…							
H. I feel children are safe crossing roads with a speed limit of…							
**Attitudes to 20mph limits**							
I. I am careful to drive at 20mph wherever limits are							
J. I understand why Edinburgh City Council introduced 20mph speed limits							
K. As long as I understand the reasons for the 20mph speed limit I will drive within the limit							
L. 20mph speed limits will take time to get used to, but eventually 20mph will be accepted as the normal speed							
M. I think 20mph speed limits will make my journeys longer							
N. It is just too difficult to stay at 20mph							
O. 20mph speed limits are a bad idea							
P. People will ignore 20mph limits because they don't see themselves getting caught by the police							
Q. I will ignore the 20mph limits if I think I will not get caught by the police							
R. If I think a road with 20mph limit has no traffic, I may drive faster than 20mph							
S. I get frustrated when I have to reduce my speed because someone in front of me is driving at 20mph							
T. I feel pressured when I drive at 20mph in a 20mph zone when the driver behind me seems annoyed							
U. I would walk more if there were more roads with 20mph speed limits							
V. I would cycle more if there were more roads with 20mph speed limits	1						
**Perceived impacts of 20mph limits**							
W. 20mph speed limits will/have made people drive slower	-0.519	1					
X. Slowly but surely 20mph limits are making me drive slower	-0.408	0.514	1				
Y. 20mph speed limits will/have led to longer journey times	0.362	-0.321	-0.275	1			
Z. 20mph speed limits will/have led to better traffic flow (less stopping and starting)	-0.532	0.506	0.457	-0.551	1		
AA. 20mph speed limits will/have led to more congestion	0.434	-0.375	-0.282	0.806	-0.563	1	
BB. 20mph speed limits will/have led to more air pollution	0.427	-0.341	-0.262	0.769	-0.499	0.792	1
CC. 20mph speed limits will/have led to decrease in fuel efficiency	0.365	-0.146	-0.239	0.534	-0.325	0.459	0.558
DD. 20mph speed limits will/have led to decreases in the number of collisions	-0.555	0.575	0.492	-0.430	0.732	-0.431	-0.418
EE. 20mph speed limits will/have led a decrease in the number of severe collisions	-0.595	0.565	0.483	-0.376	0.649	-0.378	-0.358
FF. 20mph speed limits will/have led to a n increase in people walking on the streets	-0.295	0.327	0.291	-0.216	0.451	-0.187	-0.193
GG. 20mph speed limits will/have led to an increase in people cycling	-0.545	0.476	0.398	-0.323	0.507	-0.319	-0.294

General attitudes to the road	CC	DD	EE	FF	GG
A. I use my own judgment, not speed limits, to decide on my speed on the road					
B. I tend to drive at the speed of other people on the road					
C. There is a high rate of road collisions caused by speeding in Edinburgh					
D. I feel safe walking near roads with a speed limit of…					
E. I feel safe crossing roads with a speed limit of…					
F. I feel safe cycling on roads with a speed limit of…					
G. I feel children are safe near roads with a speed limit of…					
H. I feel children are safe crossing roads with a speed limit of…					
**Attitudes to 20mph limits**					
I. I am careful to drive at 20mph wherever limits are					
J. I understand why Edinburgh City Council introduced 20mph speed limits					
K. As long as I understand the reasons for the 20mph speed limit I will drive within the limit					
L. 20mph speed limits will take time to get used to, but eventually 20mph will be accepted as the normal speed					
M. I think 20mph speed limits will make my journeys longer					
N. It is just too difficult to stay at 20mph					
O. 20mph speed limits are a bad idea					
P. People will ignore 20mph limits because they don't see themselves getting caught by the police					
Q. I will ignore the 20mph limits if I think I will not get caught by the police					
R. If I think a road with 20mph limit has no traffic, I may drive faster than 20mph					
S. I get frustrated when I have to reduce my speed because someone in front of me is driving at 20mph					
T. I feel pressured when I drive at 20mph in a 20mph zone when the driver behind me seems annoyed					
U. I would walk more if there were more roads with 20mph speed limits					
V. I would cycle more if there were more roads with 20mph speed limits					
**Perceived impacts of 20mph limits**					
W. 20mph speed limits will/have made people drive slower					
X. Slowly but surely 20mph limits are making me drive slower					
Y. 20mph speed limits will/have led to longer journey times					
Z. 20mph speed limits will/have led to better traffic flow (less stopping and starting)					
AA. 20mph speed limits will/have led to more congestion					
BB. 20mph speed limits will/have led to more air pollution					
CC. 20mph speed limits will/have led to decrease in fuel efficiency	1				
DD. 20mph speed limits will/have led to decreases in the number of collisions	-0.283	1			
EE. 20mph speed limits will/have led a decrease in the number of severe collisions	-0.226	0.767	1		
FF. 20mph speed limits will/have led to a n increase in people walking on the streets	-0.042	0.379	0.432	1	
GG. 20mph speed limits will/have led to an increase in people cycling	-0.167	0.440	0.479	0.580	1

Appendix 7 – Associations between participant demographics and travel habits, and factor scores

	i) Detraction and resistance	*Δi*	*ii) Support*	*Δii*	*iii) Rule following*	*Δiii*	*iv) Child safety*	*Δiv*	*v) Walking safety*	*Δv*
Decade of birth	▲50s, 60s, 80s<br>▼Pre-50s, 70s, 90s onwards	-	▲Younger<br>▼Older	-	▼Younger<br>▲Older	-	▲60s, 70s<br>▼Pre-50s, 50s, 80s, 90s onwards	-	▲Younger<br>▼Older	-
Gender	-	-	-	*	▲Female	-	▲Male	*	-	-
Disability	-	-	▼	-	-	*	-	-	▼	-
Ethnic minority	▼	-	-	*	-	-	-	-	-	-
SIMD16 quintile	-	*	-	-	-	-	-	-	▲Less deprived	-
Scottish Government Urban Rural Classification 2016	-	-	-	-	-	-	-	-	▲Large urban areas	-
Not working	-	-	-	*	-	-	-	*	-	-
Full UK driving license	▲	-	▼	-	▼	-	▲	-	-	-
Driving experience	-	-	-	-	▲More experience	-	▲10-40 years	-	▲5-40 years	-
Motorcycle user	▲	-	▼	-	▼	*	-	-	▼	-
Are the roads near your home or work 20mph	▲Yes<br>▼Don't know	-	-	-	-	-	-	-	-	-
Are you aware of any (more) plans for 20mph limits in the area where you live?	▲Yes<br>▼No	*	▼Yes<br>▲No	-	▼Yes<br>▲No	-	▲Yes<br>▼No	-	▼Yes<br>▲Don't know	-
Frequency of use of bus, train or tram	▼More frequent users	-	▲More frequent use	-	▲More frequent users	-	▼Regular users	-	▲Regular users	-
Frequency of use of a car or van	▲More frequent users	-	▼More frequent users	-	▼More frequent users	*	▲More frequent users	-	▲Regular users	-
Frequency of use of taxi or Uber	-	-	-	-	▼More frequent users	-	-	-	▲Regular users	-
Cycling frequency	▼More frequent users	-	▲More frequent users	-	▲More frequent users	-	▲Regular users	-	▲Regular users	-
Walking or running frequency	▼More frequent users	-	▲More frequent users	-	▲More frequent users	*	-	-	▲Regular users	*

Δ; change in listed factor over time, -; not statistically significant (p>0.05), ▲; statistically significant increase in scores based on a Kruskal-Wallis test, ▼; statistically significant decrease in scores based on a Kruskal-Wallis test, *; the change in factor scores over time varied statistically significantly by this variable

Appendix 8 – Associations between participant demographics and travel habits, and change in factor scores

Detraction and resistance

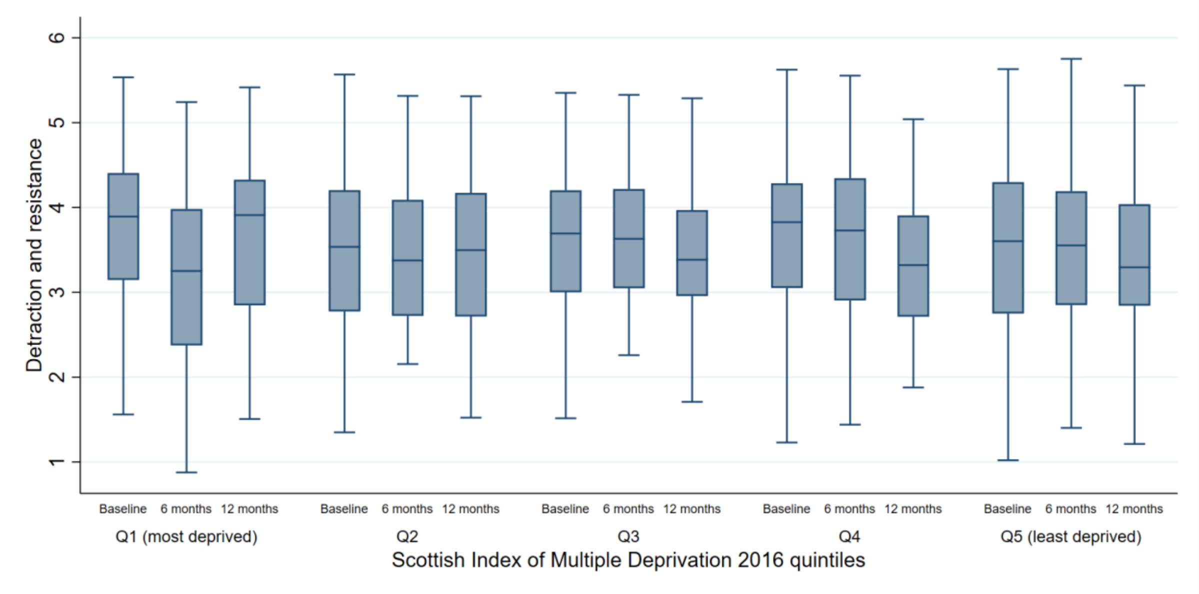

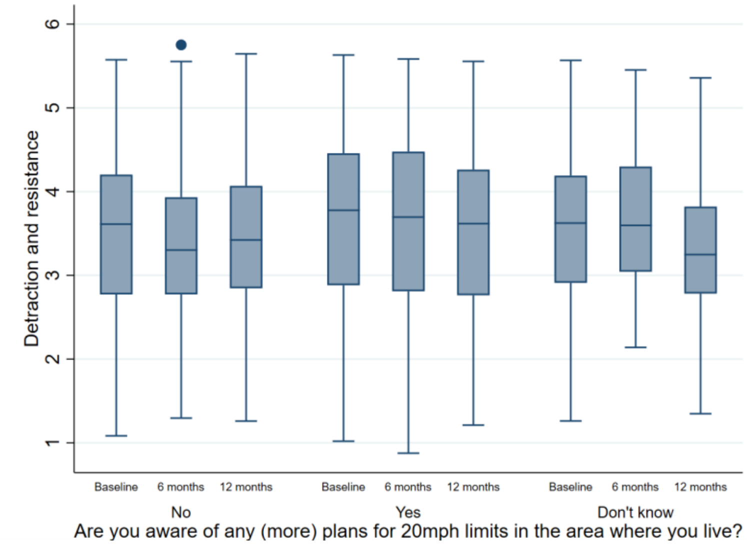

Support

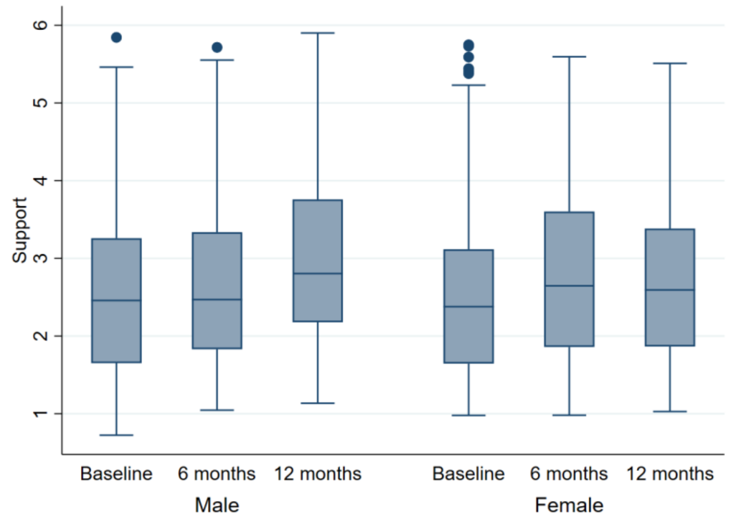
Support
1
2
3
4
5
6
Baseline
6 months
12 months
Baseline
6 months
12 months
Male
Female

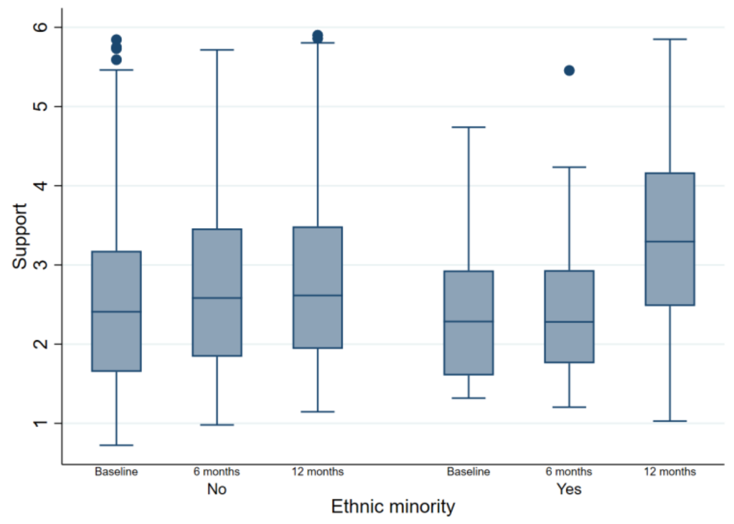
Support
1
2
3
4
5
6
Baseline
6 months
12 months
Baseline
6 months
12 months
No
Yes
Ethnic minority

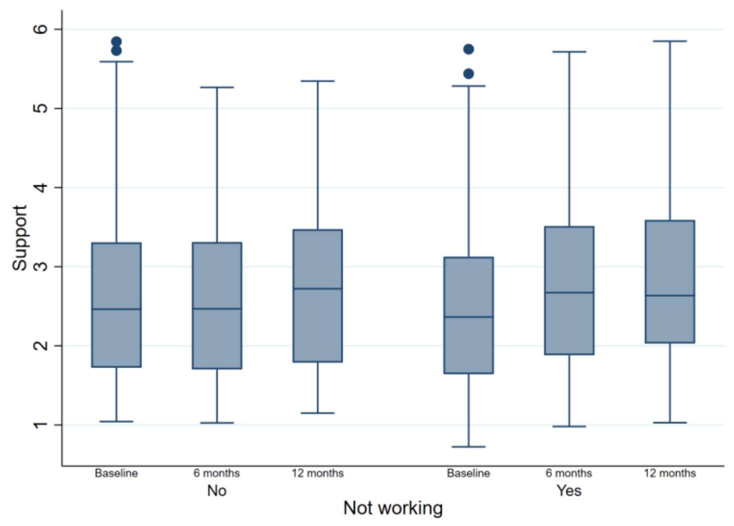
Support
1
2
3
4
5
6
Baseline
6 months
12 months
Baseline
6 months
12 months
No
Yes
Not working

Rule following

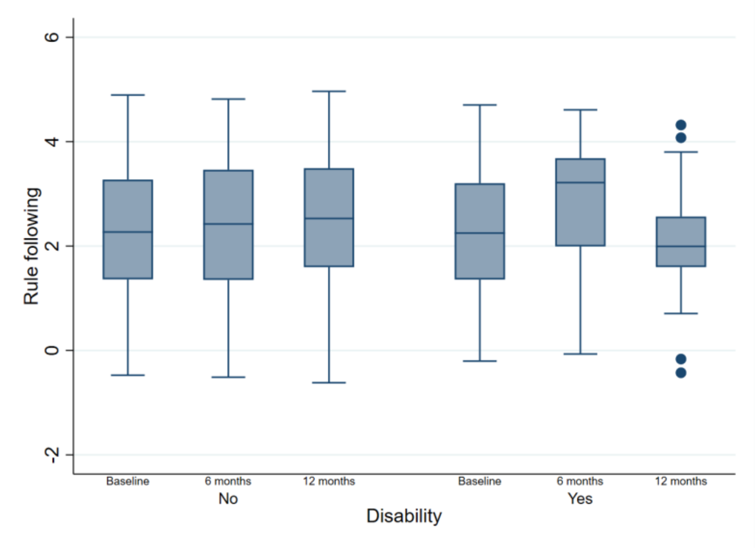

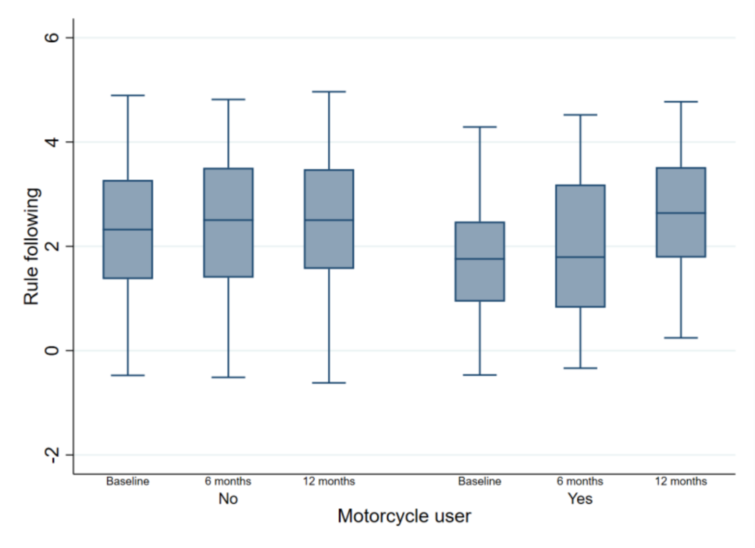

Rule following (cont.)

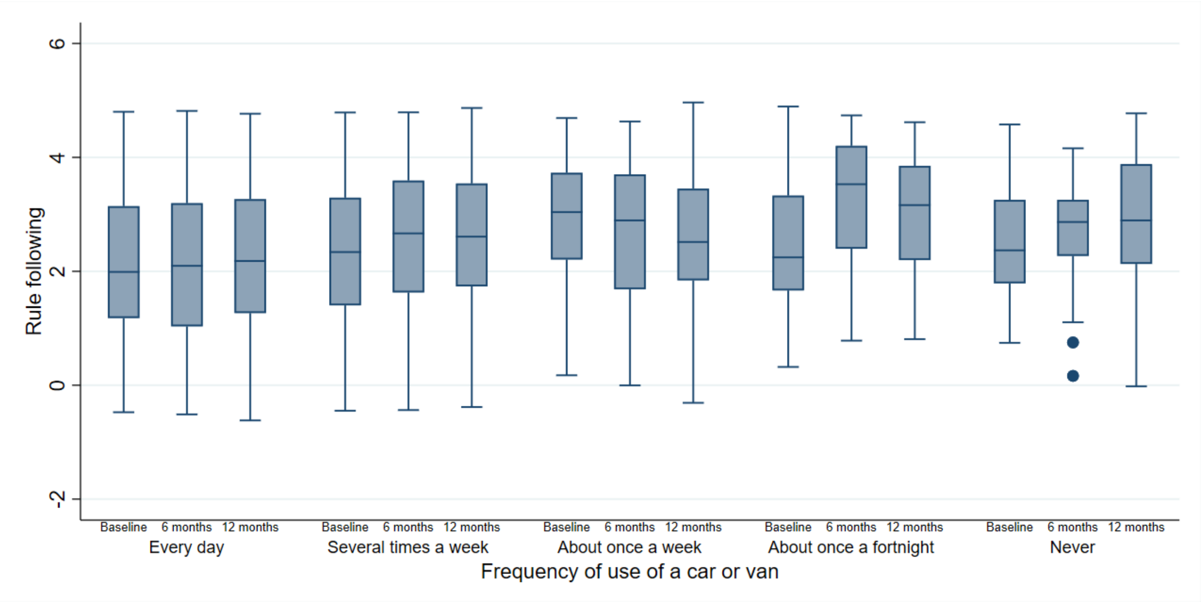

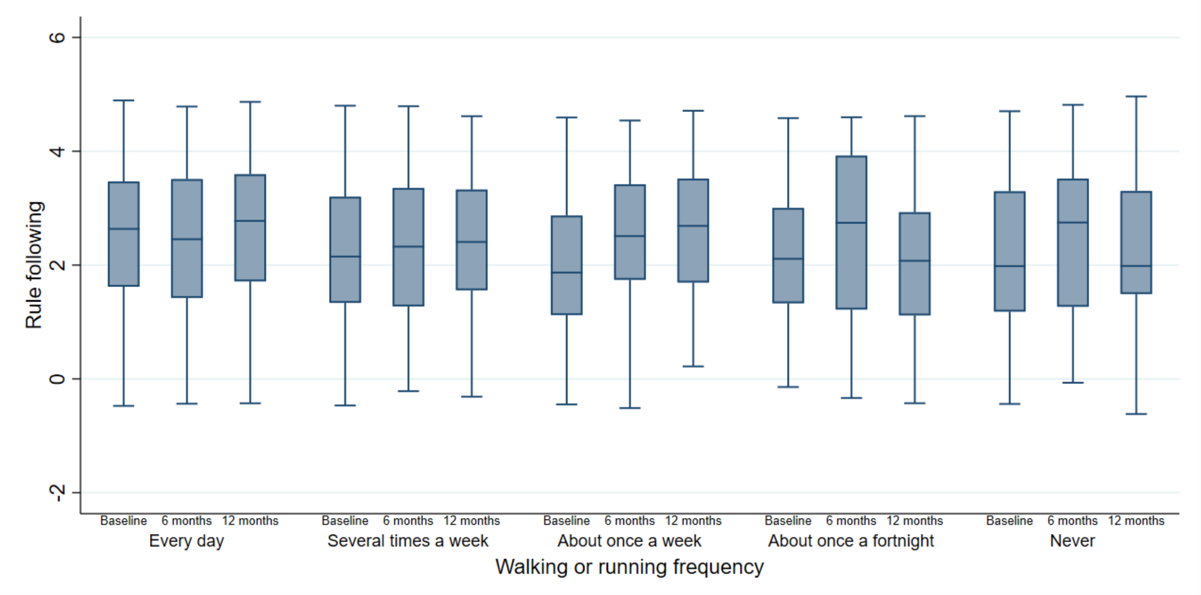

Child safety

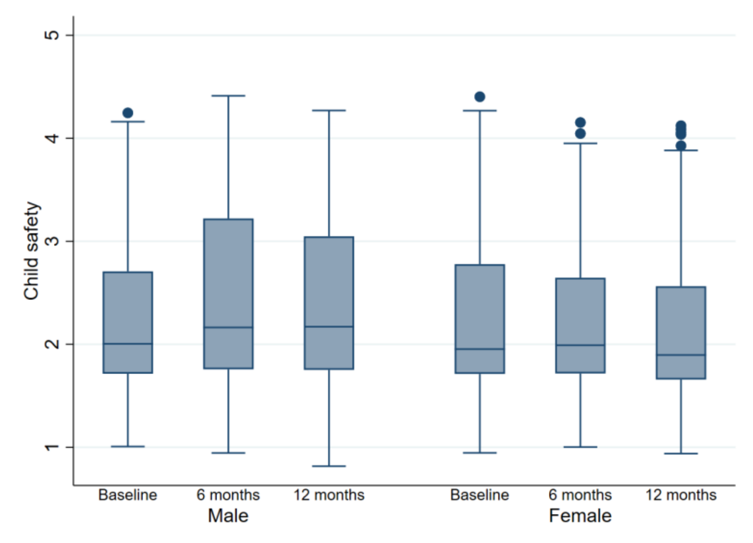

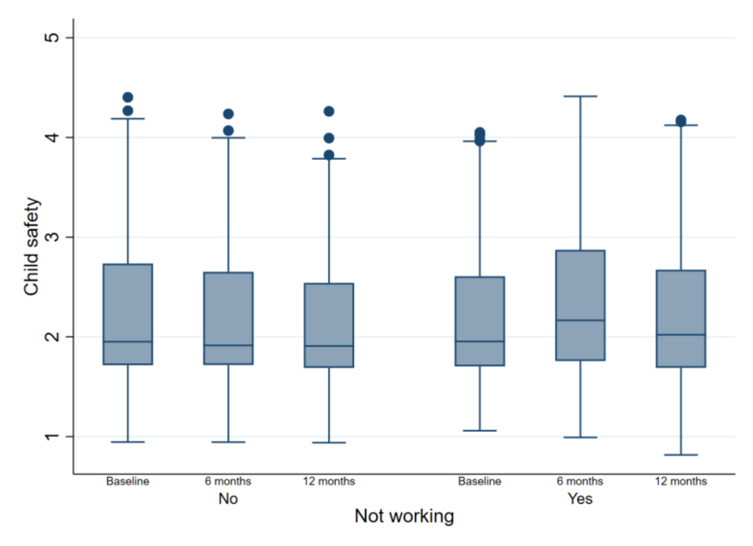

Walking safety

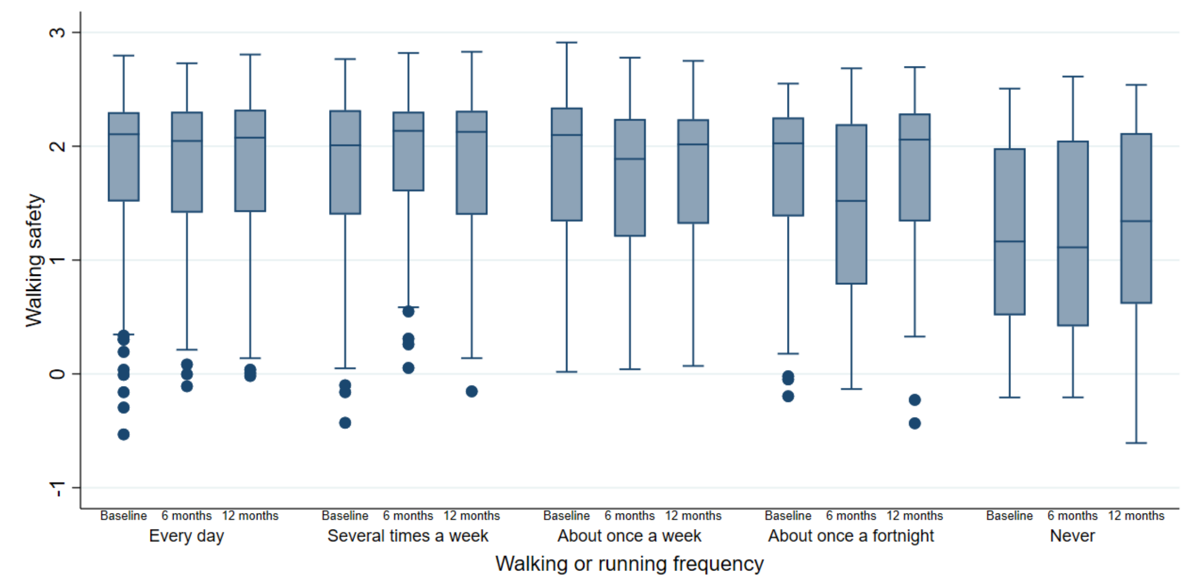

Supplement: Supplementary data 1 [file mmc1.pdf]
